# Understanding the consequences of leisure sedentary behavior on periodontitis: A two-step, multivariate Mendelian randomization study

**DOI:** 10.1016/j.heliyon.2023.e23118

**Published:** 2023-11-30

**Authors:** Zhonghua Zhang, Ming Ding, Hui Ding, Yuyan Qian, Jiaxing Hu, Jukun Song, Zhu Chen

**Affiliations:** a School of Stomatology, Zunyi Medical University, Zunyi, China; b Department of Endodontics, Guiyang Stomatological Hospital, Guiyang, China; c Department of Oral and Maxillofacial Surgery, The Affiliated Stomatological Hospital of Guizhou Medical University, Guiyang, China; d Department of Neurosurgery, The First Affiliated Hospital of Hainan Medical College, Haikou, China

**Keywords:** Leisure sedentary behavior, Mendelian randomization, Periodontitis, Smoking, Intermediary analysis

## Abstract

**Background:**

The relationship between leisure sedentary behavior (LSB) and periodontitis risk remains unclear in terms of causality and the potential mediating effects of intermediate factors.

**Materials and methods:**

Using the aggregate data of several large-scale genetic association studies from participants of European descent, we conducted a univariate, two-step, and multivariate Mendelian random (MR) analysis to infer the overall effect of LSB on periodontitis, and quantified the intermediary proportion of intermediary traits such as smoking.

**Results:**

Our findings indicated that per 1-SD increase (1.87 h) in leisure screen time (LST), there was a 23 % increase in the risk of periodontitis. [odds ratios (95 % CI) = 1.23 (1.04–1.44), p = 0.013]. Smoking was found to partially mediate the overall causal effect of LST on periodontitis, with a mediation rate of 20.7 % (95 % CI: 4.9%–35.5 %). Multivariate MR analysis demonstrated that the causal effect of LST on periodontitis was weakened when adjusting for smoking, resulting in an odds ratio of 1.19 (95 % CI: 1.01–1.39, p = 0.049) for each 1 standard deviation increase in exposure.

**Conclusion:**

The study provides evidence of a potential causal relationship between LSB characterized by LST and periodontitis, thereby further supporting the notion that reducing LSB is beneficial for health. Furthermore, it confirms the role of smoking as a mediator in this process, suggesting that inhibiting smoking behavior among individuals with long-term LSB may serve as a strategy to mitigate the risk of periodontitis.

## 1 Introduction

Periodontitis, a chronic multi-factor inflammatory disease of periodontal supporting tissue, is the product of the interaction among pathogenic microorganisms, host response, and personal health habits [1]. The global oral disease survey conducted by Marco et al. reveals that periodontitis, ranking sixth among the world's diseases, impacts 10.8 % of the global population, which amounts to over 743 million individuals [1]. As a result, periodontitis should be recognized as a significant global public health challenge, necessitating the implementation of appropriate preventive measures to alleviate the future medical burden.

Leisure sedentary behavior (LSB) refers to any awake behavior characterized by the energy consumption of less than 1.5 metabolic equivalents in sitting or reclining posture [2]. At present, a large number of studies have shown that LSB, such as television watching, computer use, and driving, may lead to coronary artery disease, cancer, and even an increase in all-cause mortality [3,4]. LSB is closely associated with periodontitis. A recent cross-sectional study using data from the NHANES database found that prolonged LSB exceeding 7.5 h per day was linked to an increased risk of periodontal disease [5]. Nevertheless, due to the absence of prospective studies and the interference of confounding factors, establishing a causal relationship between LSB and periodontitis in current observational research is challenging. The mechanism by which LSB contributes to the development of periodontitis is also not fully understood.

Mendelian randomization (MR) is based on the principle of random allocation of alleles during gametogenesis, replicating the randomization process of randomized controlled trials. It infers the causal relationship between exposure and outcome by utilizing genetic variation as an instrumental variable (IV), effectively mitigating confounding biases in traditional epidemiological studies [6]. The widely publicized genome-wide association study (GWAS) provides reliable analytical data for MR. This study utilizes a large-scale GWAS of sedentary behavior, encompassing activities like leisure screen time (LST), sedentary behavior at work, and sedentary commuting, to perform a univariate MR analysis and explore the causal role of LSB in periodontitis. At the same time, this study also introduced two-step MR and multivariate MR for intermediary analysis to decipher whether there is a potential intermediary pathway between LSB and periodontitis (such as body mass index (BMI), smoking, and drinking).

## 2 Materials and methods

### 2.1 Research and design

In this study, we extracted IVs from the GWAS of LSB (including LST, sedentary behavior at work, and sedentary commuting), and used univariate two-ample MR analysis to study the causal effect of LSB on periodontitis risk. Two-step MR was used to evaluate the effects of candidate intermediary traits (including smoking, alcohol consumption, BMI, three body fat distribution traits, five lipid traits, and four blood glucose traits) on the causal relationship between LST and periodontitis, and multivariate MR was used for secondary verification. Finally, we used the analytical data obtained from two-step MR to conduct an intermediary analysis to estimate the impact of LST mediated by smoking on periodontitis.

### 2.2 Data source

The summary-level data for LST, sedentary behavior at work, and sedentary commuting were sourced from a meta-analysis of whole-genome studies involving 526,725 individuals, 372,605 individuals, and 159,606 individuals of European ancestry [7]. These three sedentary behaviors were measured using self-reported data specific to the respective domains and intensity. LST included time spent watching television, playing video games, sitting at the computers, etc. And it was treated as a continuous variable with a mean (Standard deviation, SD) of 3.53 (1.87) hours per day. After harmonizing the phenotypes from 34 to 15 different surveys included in the respective studies, sedentary behavior at work and sedentary commuting were defined as binary variables representing being in a sedentary state or not. Sedentary behavior at work referred to mostly sitting and no heavy lifting, while sedentary commuting indicated driving a car during the commute. Each study conducted GWAS analyses stratified by gender, adjusting for age, genetic principal components, and study-specific covariates.

The summarized data on periodontitis was obtained from the Gene-Lifestyle Interactions in Dental Endpoints (GLIDE) consortium. These cases originated from seven cohorts that contributed to the GLIDE consortium. The inclusion criteria for PD cases in this study were as follows: four studies used the definitions provided by the Centers for Disease Control and Prevention/American Academy of Periodontology for diagnosis, two studies were confirmed based on similar criteria evaluated through probing depth, and one study relied on self-reported diagnoses. After excluding the Hispanic Community Health Study/Latino Study (HCHS/SOL) cohort to ensure a European sample, a total of 12,289 cases and 22,326 controls were included [8]. To improve the credibility and universality of the study, we also selected the R8 version of periodontitis GWAS (N example = 2118 N control = 340,381) from the FinnGen for replication verification. K05.31 in the revised International Classification of Diseases (ICD-10) code defines the diagnostic criteria for periodontitis (https://www.finngen.fi/en/access_results) [9].

Regarding the mediator traits used for the two-step MR, we selected some candidate mediator traits as potential mediators of television watching responsibility, including BMI, smoking, drinking, 3 body fat distribution traits [waist circumference（WC）, hip circumference (HIP), waist-to-hip ratio (WHR)],5 lipid traits [including high-density lipoprotein (HDL) cholesterol, low-density lipoprotein (LDL) cholesterol, triglyceride (TG), total cholesterol (TC)] and 4 blood glucose traits [including fasting glucose (FG), fasting insulin (FI), 2-h postprandial glucose (2 hGlu), hemoglobin A1c (HbA1c)]. The GWAS statistical data for BMI, smoking, drinking, lipid traits, and blood glucose traits were collected from the Genetic Investigation of Anthropometric Traits (GIANT) Alliance, the GWAS & Sequencing Consortium of Alcohol and Nicotine use (GSCAN), the Meta-Analyses of Glucose and Insulin-related traits Consortium (MAGIC) and the Global Lipids Genetics Consortium Results (GLGC). Except for the traits of smoking, BMI, and drinking, the data on the above mediator traits excluded the population from the UK Biobank. The original literature corresponding to the five mediated traits can be found here [[10], [11], [12], [13], [14]].

The above GWAS data are all from the European population. More information about the source of the data can be obtained in Table S1. The MR study used publicly shared data sets. These data sets were approved by the relevant ethics committees and agreed upon by all participants in the original GWAS study and its contribution cohort. Therefore, no additional moral declaration or consent is required.

### 2.3 Instrumental variable selection

MR is a method of exploring causality through IVs. Single nucleotide polymorphism（SNP） is usually used as a genetic IV. MR design must meet three assumptions: (i) genetic IVs are closely related to exposure; (ii) genetic IVs are independent of potential confounding factors, and (iii) genetic IVs affect outcomes only through risk factors [15]. To select an effective IV that meets the above three assumptions, we have taken a series of measures. First, select the SNPs that are closely related to exposure (P < 5 × 10–8). Secondly, the SNPs in strong linkage disequilibrium（LD） were excluded (LD R2 < 0.001, LD distance >10,000 kb). Third, the PhenoScanner V2 （http://www.phenoscanner.medschl.cam.ac.uk/） database is used to detect the potential multiplicity of SNPs. SNPs identified as associated with potential confounding factors were excluded from the analysis. Finally, SNPs with inconsistent allele direction between exposure and outcome were excluded to avoid reverse causality.

In addition, we also calculate the F statistics of each SNP by the following equation: $F=\frac{N-k-1}{k}\times \frac{R^{2}}{1-R^{2}}$ (N is the number of samples, k is the number of IV, and R^2^ refers to the genetic instrument set used to explain the degree of variation of traits). Since R^2^ is not usually provided in the GWAS summary data, we use the formula $\sum \left[\frac{\left(\beta^{2}\cdot 2\cdot f\cdot \left(1-f\right)\right)}{\left(\beta^{2}\cdot 2\cdot f\cdot \left(1-f\right)+se^{2}\cdot 2\cdot n\cdot f\cdot \left(1-f\right)\right)}\right]$ to calculate R^2^ (f is the effect allele frequency, n is the sample size, β is the effect estimate for each SNP, and se is the standard error for each SNP). IV with F statistics less than 10 is considered to be a weak IV and will be excluded from MR analysis.

## 3 Statistical analysis

### 3.1 Mendelian randomized analysis

**Fig. 1:**
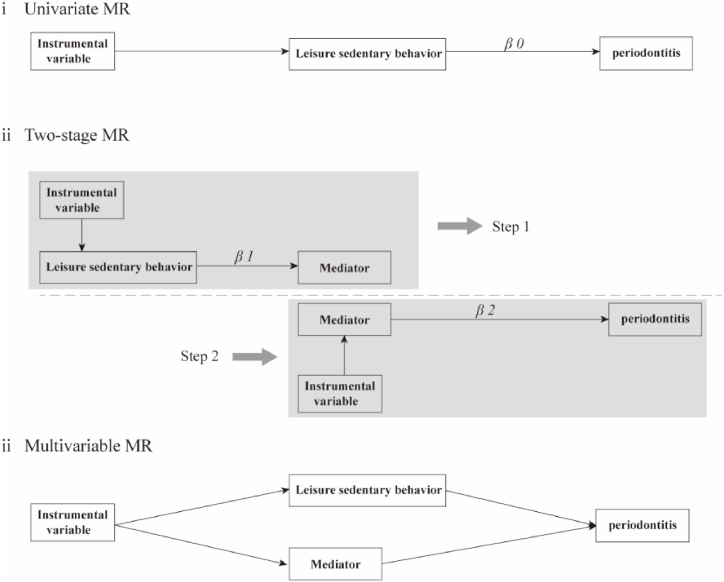
I: Schematic diagram of univariate Mendelian randomization principle; β0: total causal effect value of leisure sedentary behavior on periodontitis. **ii:** Schematic diagram of two-step Mendelian randomization principle; **β1:** causal effect value of leisure sedentary behavior on mediating traits. **β2:** Causal effect values of mediating traits on periodontitis. **iii:** Schematic diagram of Multivariable Mendelian randomization principle.

We conducted univariate Mendelian randomization (UVMR) analysis to investigate the causal relationship between sedentary behavior and periodontitis **[**Fig. 1(i)**]**. Multiple methods were utilized to estimate the causal effects of exposure on outcomes, including random effects inverse variance weighted (IVW) [16], MR-Egger [17], weighted median [18], MR using a Robust Adjusted Profile Score (MR-raps) [19], and Mendelian Randomization Pleiotropy RESidual Sum and Outlier (MR-PRESSO) [20]. These analytical methods exhibit variations in terms of their hypotheses on multiplicity and the statistical efficiency of genetic tools. The advantages and disadvantages of these different methods are detailed in Table S2. Due to its superior statistical efficiency and widespread usage, IVW is considered the primary analysis method. Non-significant results (p > 0.016) from other methods that align with the direction of the MR-IVW estimated effect, in the absence of heterogeneity and pleiotropy, are also considered supportive [[21], [22], [23]]. The causal effect obtained at this stage is the overall effect of LSB on periodontitis, which we denote as β0. The Bonferroni-corrected significance threshold was established at 0.016 to accommodate the impact of multiple testing, and a lenient threshold P < 0.05 was considered to suggest a causal relationship.

Mediation analysis is a method aimed at exploring how exposure affects outcomes. Mediation analysis breaks down the total effect of exposure on outcomes into direct effects (effects not mediated by mediation) and indirect effects (effects mediated by mediation). The total effect can be obtained from the UVMR（β0）. For indirect effects, we choose to obtain them through two-step MR.

The two-step MR is essentially composed of two independent univariable two-sample MR analyses **[**Fig. 1**(ii)]**. In step one, LBS was used as exposure and intermediate traits as an outcome; Note the causal effect size obtained in this step as β1. The reverse causality between LST and each mediator trait was assessed using UVMR to eliminate bidirectionality that could impact the validity of the mediation model. In step 2, media was used as exposure, and periodontitis as a result. The causal effect size obtained in this step is denoted as β2. It is important to note that the IVs used in Step 2 should exclude the IVs used in Step 1.

The indirect effect of each mediator (β1*β2) is obtained by multiplying β1 with β2 by the production method. The mediation ratio of each mediator was quantified by dividing the indirect effect by the total effect (β1*β2/β0). The Delta method is selected as the estimation technique of the corresponding confidence interval [24].

Multivariate MR is used to verify the selected intermediate traits **[**Fig. 1**(iii)]**. Several different methods were adopted to conduct multivariable MR, including random effects IVW, MR-Egger, MR-Lasso, and MR-Presso. IVW was also used as the primary analysis method. By checking whether the causal effect size of the outcome is impaired by adjusting the exposure of a mediating trait, we can verify whether the mediating trait mediates the process of exposure and outcome.

### 3.2 Sensitivity analysis

First, we examined heterogeneity between causal estimates of individual SNPs by the IVW and MR-Egger methods using Cochran's Q statistic. Funnel plots are also used as a complementary method to detect heterogeneity because we can intuitively judge whether there is obvious heterogeneity from the degree of symmetry of its graph. Second, to test for horizontal pleiotropy, we calculated the intercept of the MR-Egger regression line and applied MR-PRESSO (pleiotropic residuals and outliers) to detect any horizontal pleiotropic outliers [[24], [25], [26]]. The leave-one-out method was also used to test the robustness of the MR analysis results, which was to eliminate each SNP one by one and observe whether the results obtained before and after the elimination were statistically different.

Additionally, we computed F-statistics for all traits related to sedentary behavior by applying the formula specified in the instrumental variable screening module. Power was estimated using an online tool (https://shiny.cnsgenomics.com/mRnd/).

All MR analyses were conducted using R (version 4.2.1) through “TwoSampleMR (version 0.5.6)”, “MRPRESSO (version 1.0)”, “MRRAPS (version 0.4)”, and “MendelianRandomization (version 0.5.1)” R packages.

## 4 Result

### 4.1 Univariate mendelian randomization analysis

In the MR Study with GLIDE alliance periodontitis data as the outcome, 92,7 and 13 SNPs were selected as IVs for three phenotypes of LSB. In a replication study using a FinnGen database of periodontitis outcomes, 91,7 and 13 SNPs were selected as IVs for three phenotypes of LSB. The IVs acquisition process is shown in Fig. S1. The F statistics of the SNPs screened above are all greater than 20 (Table S3), which indicates that our MR is unlikely to be affected by weak instrumental variable bias.

The results of the UVMR analysis showed a positive association between genetically predicted LST and the risk of periodontitis among the three sedentary behaviors [OR (95 % CI) = 1.23 (1.04, 1.44), P = 0.013], with no heterogeneity (P _MR–IVW Heterogeneity_ = 0.69) or pleiotropy detected (P_MR–Egger Intercept_ = 0.80, P_Global Test_ = 0.42). Specifically, our findings indicated that for each 1-SD (1.87 h) increase in LST, there was a 23 % increase in the risk of periodontitis (Table 1). The sufficient statistical power of our study makes it less likely to be affected by type II errors (Table S4). This positive correlation was replicated in the independent dataset (Table 2). The associations remained consistent in sensitivity analyses**（**Fig. S2**-S19）**

**Table 1:** UVMR analysis for the association of genetically predicted leisure sedentary behavior on periodontitis from the GLIDE Alliance.

Exposure	Outcome	Nsnp	Method	beta	OR	95%CI	p_value	Heterogeneity P_value	Intercept_P_value	Global Test
**leisure screen time**	**PD**	92	**IVW**	0.20	1.23	(1.04, 1.44)	**0.013**	0.69	NA	NA
			**MR–Egger**	0.30	1.35	(0.64, 2.85)	0.433	0.72	0.80	NA
			**WM**	0.23	1.26	(1.01, 1.58)	**0.044**	NA	NA	NA
			**MR-RAPS**	0.16	1.17	(1.01, 1.36)	**0.046**	NA	NA	NA
			**MR-PRESSO**	0.15	1.16	(1.01, 1.35)	**0.048**	NA	NA	0.42
**sedentary behavior at work**	**PD**	7	**IVW**	0.25	1.29	(0.84, 1.98)	0.252	0.34	NA	NA
			**MR–Egger**	0.95	2.58	(0.58, 11.40)	0.268	0.35	0.38	NA
			**WM**	0.37	1.45	(0.84, 2.50)	0.185	NA	NA	NA
			**MR-RAPS**	0.25	1.28	(0.88, 1.87)	0.190	NA	NA	NA
			**MR-PRESSO**	0.25	1.28	(0.92, 1.79)	0.186	NA	NA	0.38
**sedentary commuting**	**PD**	13	**IVW**	0.11	1.12	(0.82, 1.53)	0.482	0.22	NA	NA
			**MR–Egger**	−0.11	0.89	(0.40, 1.99)	0.786	0.26	0.56	NA
			**WM**	−0.01	0.99	(0.67, 1.45)	0.951	NA	NA	NA
			**MR-RAPS**	0.08	1.08	(0.82, 1.42)	0.576	NA	NA	NA
			**MR-PRESSO**	0.07	1.08	(0.82, 1.42)	0.609	NA	NA	0.34

**Abbreviations: Nsnp:** Number of SNPs; **OR:** Odds ratio; **CI:** Confidence interval; **IVW:** Inverse-variance weighted**; WM:** Weighted median; **MR-RAPS:** Mendelian Randomization using a Robust Adjusted Profile Score; **MR-PRESSO:** Mendelian Randomization Pleiotropy RESidual Sum and Outlier; **PD:** Periodontitis.

**Table 2:** UVMR analysis for the association of genetically predicted leisure sedentary behavior on periodontitis from the FinnGen database.

Exposure	Outcome	Nsnp	Method	beta	OR	95%CI	p_value	Heterogeneity P_value	Intercept_P_value	Global Test
**leisure screen time**	**PD**	91	**IVW**	0.31	1.37	(1.04, 1.80)	**0.025**	0.81	NA	NA
			**MR–Egger**	1.40	4.06	(1.13, 14.57)	**0.035**	0.76	0.09	NA
			**WM**	0.43	1.53	(1.03, 2.28)	**0.034**	NA	NA	NA
			**MR-RAPS**	0.20	1.22	(1.03, 1.45)	**0.025**	NA	NA	NA
			**MR-PRESSO**	0.19	1.2	(1.01, 1.44)	**0.046**	NA	NA	0.588
**sedentary behavior at work**	**PD**	7	**IVW**	0.52	1.68	(0.67, 4.25)	0.270	0.08	NA	NA
			**MR–Egger**	1.07	2.91	(0.08, 101.04)	0.582	0.13	0.77	NA
			**WM**	0.63	1.88	(0.68, 5.16)	0.221	NA	NA	NA
			**MR-RAPS**	0.42	1.52	(0.79, 2.91)	0.209	NA	NA	NA
			**MR-PRESSO**	0.41	1.5	(0.72, 3.13)	0.308	NA	NA	0.253
**sedentary commuting**	**PD**	13	**IVW**	0.24	1.28	(0.74, 2.20)	0.382	0.26	NA	NA
			**MR–Egger**	0.35	1.42	(0.39, 5.21)	0.606	0.33	0.86	NA
			**WM**	0.32	1.38	(0.65, 2.93)	0.405	NA	NA	NA
			**MR-RAPS**	0.22	1.24	(0.75, 2.04)	0.396	NA	NA	NA
			**MR-PRESSO**	0.21	1.23	(0.77, 1.97)	0.403	NA	NA	0.471

**Abbreviations: N_snp:** Number of SNP; **OR:** Odds ratio; **CI**, Confidence interval.

However, we did not observe a significant causal effect of sedentary behavior at work and sedentary commuting on periodontitis (Table 1, Table 2). Because of shared biological pathways, periodontitis may further influence unhealthy lifestyles. To explore whether there is a reverse causality, we performed a reverse MR Analysis. We did not observe any significant causal association between genetic susceptibility to periodontitis and the three LSBs (Table S5, all P > 0.05).

In conclusion, the UVMR suggests a potentially causal relationship between LST and increased risk of periodontitis.

### 4.2 Two-step mendelian randomization

**Fig. 2:**
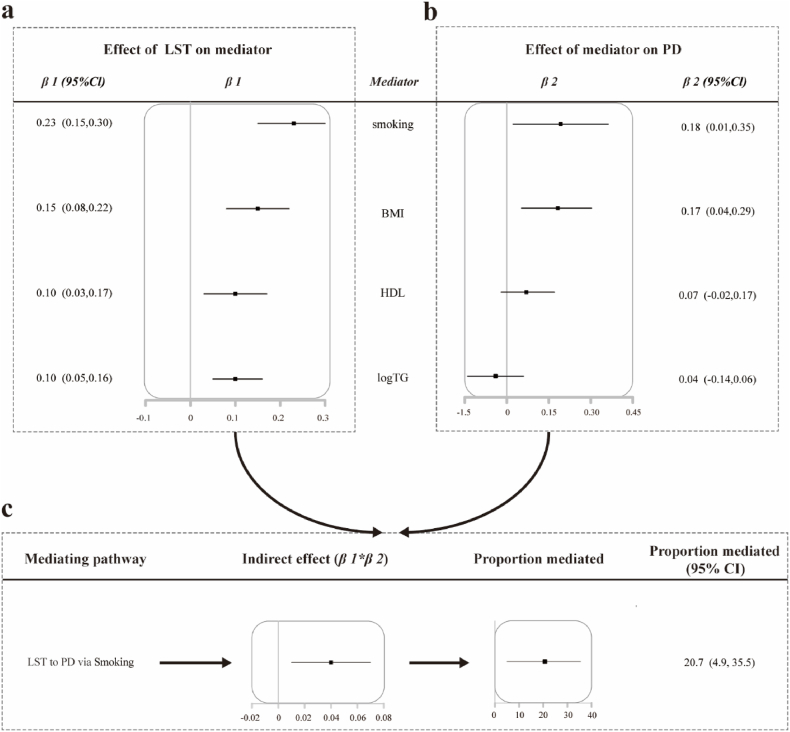
Two-step MR mediation analysis of the association between LST and Periodontitis. **Note:** The IVW causal effect size is the beta coefficient estimated by the IVW model for the corresponding outcome; Mediated proportion = indirect causal effect of coefficient product/total causal effect (β1*β2/β0).

The two-step MR analysis revealed the causal effects of smoking and BMI in both steps **[**Fig. 2(a**-b)]**. Wever, in the reverse MR analysis conducted in the first stage, we found bidirectional causal associations between LST and BMI, while a unidirectional causal relationship existed between LST and smoking (Table S5). Due to the limitations of existing IV mediation methods in estimating mediation under exposure-mediator interaction, we excluded the mediation effect of BMI [[27], [28], [29]]. The mediation analysis revealed that smoking partially mediated the causal relationship between LST and periodontitis [mediated proportion (95%CI) = 20.7 % (4.9 %, 35.5 %)]. It is important to note that only mediator traits that demonstrate a causal effect in the first step and pass the sensitivity analysis can be selected for the second step of two-step Mendelian randomization. Fig. 2(a-c) sualizes the results of the two-step Mendelian randomization (MR) analysis using IVW as the main analytical method, as well as the results of the mediation analysis. Detailed results of the two-step MR analysis for all mediator traits can be found in Table S6.

### 4.3 Multivariable Mendelian randomization

**Fig. 3:**
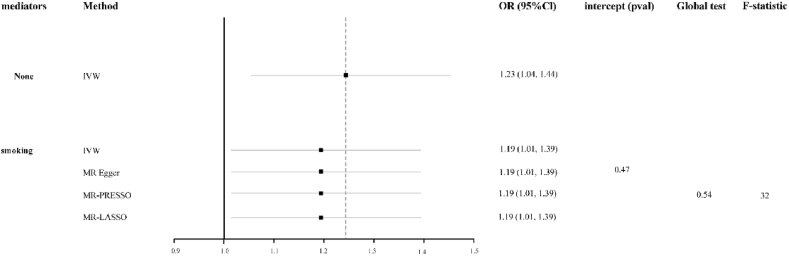
Multivariate MR analysis of the causal relationship between LST and periodontitis after adjusting smoking **Abbreviations: IVW:** Inverse-variance weighted; **OR:** odds ratio; **CI:** confidence interval**.**

To increase the reliability of the results, we employed multivariable MR to validate the selected mediator (smoking) mentioned above. The results indicated that the causal effect of LST on periodontitis was weakened when adjusting for smoking, resulting in an odds ratio of 1.19 (95 % CI: 1.01–1.39, p = 0.049) for each 1 standard deviation increase in exposure.（Fig. 3）

A series of sensitivity analyses showed that the genetic instrumental variables exhibited no heterogeneity, no pleiotropy, and had sufficient statistical power (P_MR–Egger Intercept_ = 0.47, P_Global_ _Test_ = 0.54， F-statistic = 32).

## 5 Discussion

Utilizing large-scale genetic association data within the framework of MR, we identified a potential causal relationship between LST (a long time presenting Leisure sedentary behavior) and periodontitis. Moreover, a two-step MR analysis demonstrated that smoking partially mediates the association between LST and periodontitis. Multivariate MR again validated the mediating role of smoking.

These findings are consistent with previous research. An observational study found a correlation between LSB and a higher prevalence of periodontal disease [5]. A systematic review demonstrated that the frequency of physical activity was directly associated with a reduced incidence of periodontitis [30]. LSB, as a typical low-frequency physical activity behavior, may be more likely to cause periodontitis. However, a review of previous literature found that LSB does not directly endanger health but often mediates the occurrence of diseases (including lung cancer and diabetes) through intermediary traits such as obesity and smoking [31,32]. An observational study identified a positive relationship between smoking status and increased TV viewing time [33]. The detrimental effects of smoking on periodontitis have been extensively documented. A systematic review of 238 prospective studies confirmed that smoking increases the risk of developing periodontitis. Recent evidence from MR analysis has further strengthened this causal relationship [34]. Some scholars postulate that smoking may influence the immune response and the healing capacity of periodontal tissue, resulting in periodontitis [35,36]. Moreover, our study observed the potential mediating role of BMI, which is consistent with previous findings demonstrating that advertisements and food cues during recreational screen activities, like watching TV, can lead to higher food intake and overall energy consumption, contributing to obesity [37,38]. Obesity is likewise recognized as a significant risk factor for periodontitis [39]. However, due to the bidirectional causal association between recreational LST and BMI, it is challenging to determine whether BMI acts as a mediator or a confounding factor, even with the employment of IV mediation analysis. Thus, we exercise caution when interpreting the mediating role of BMI in this context.

It is important to note that even after controlling for smoking, there remains a causal relationship between LST and periodontitis. This suggests the presence of unexplored mediating factors. Further research is needed to fully investigate the mediating traits that underpin this process.

Using the MR method, we did not discover a significant association between sedentary behavior at work and sedentary commuting and the occurrence of periodontitis. Limited studies exist on the distinctions among the three types of LSB and their relationship with periodontitis. Nevertheless, certain epidemiological evidence might offer insights into this matter. LST is more likely to be associated with behaviors such as diet and smoking, compared to sedentary behavior at work and sedentary commuting [33,37]. These behaviors are already recognized as contributors to periodontitis.

Our study possesses multiple strengths. Firstly, it represents the inaugural MR analysis investigating the association between LSB and periodontitis. Drawing on conventional MR research, we further selected 15 mediator traits as candidates for mediation MR. Moreover, we conducted a secondary validation of the mediator traits identified in the two-step MR using multivariate MR, thereby enhancing result reliability. Lastly, we conducted sensitivity analyses at each stage of the analysis to minimize potential biases in our study.

However, this study still has some limitations. The UVMR analysis of sedentary behavior at work, sedentary commuting, and periodontitis had limited statistical power due to inadequate estimation of heritability from the selected SNPs in the exposure data. Therefore, we approach the non-causal relationship between sedentary behavior, commuting to work, and periodontitis with caution, recognizing the need for further research with additional GWAS data related to LSB. Secondly, during the UVMR phase, although the initial analysis's IVW estimate passed correction for Bonferroni (P < 0.016), the lack of statistical significance in other MR estimation methods under Multiple hypothesis testing correction limitations constrained the strength of the causal relationship. Similarly, in the replication validation using data from FinnGen, the IVW estimation did not pass the more stringent Bonferroni correction. This outcome could potentially be attributed to the smaller number of cases/controls in the FinnGen database (2118/340,381) compared to the case/control ratio in the GLIDE consortium (12,289/22,326), thereby impacting the level of statistical significance. Conducting large-scale GWAS will be necessary to ascertain the robustness of the causal effects. Furthermore, this study's data is limited to European populations, and whether this conclusion can be extrapolated to other ethnic groups requires further investigation.

## 6 Conclusion

Our MR results provide suggestive evidence for a causal relationship between leisure sedentary behavior characterized by leisure screen time and periodontitis. This finding further strengthens the concept that reducing sedentary behavior is beneficial for health. Additionally, the results demonstrate a mediating effect of smoking, suggesting that inhibiting smoking behavior among individuals with long-term leisure sedentary behavior may serve as a strategy to mitigate the risk of periodontitis.

## Funding information

This work was supported by the Science and Technology Fund of the Guizhou Provincial Health Commission (gzwkj2023-204 and gzwkj2021-341).

## Data availability statement

Leisure sedentary behavior data are available at https://www.ebi.ac.uk/gwas/. Periodontitis based data is available at https://data.bris.ac.uk/data/dataset/2j2rqgzedxlq02oqbb4vmycnc2 and https://r8.finngen.fi/. Three types of Body fat distribution (waist circumference, chest circumference, waist-to-hip ratio) data can be accessed athttps://portals.broadinstitute.org/collaboration/giant/index.php/GIANT_consortium_data_files. Five lipid traits (high-density lipoprotein cholesterol, low-density lipoprotein cholesterol, triglyceride, total cholesterol) are available at https://csg.sph.umich.edu/willer/public/glgc-lipids2021/. Four blood glucose traits (fasting glucose, fasting insulin, 2-h postprandial glucose, hemoglobin A1c) can be assessed at https://magicinvestigators.org/. Smoking and drinking in the data are available at https://www.thessgac.org/data.

## Ethics statementstatement

Each of the studies contributing to the GWAS meta-analyses obtained informed consent from the study participants. This study complied with all relevant ethical regulations, including the Declaration of Helsinki, and ethical approval for data collection and analysis was obtained by each study from local boards as described in the included GWAS.

## Additional information

No additional information is available for this paper.

## CRediT authorship contribution statement

**Zhonghua Zhang:** Writing – original draft, Visualization, Software, Methodology, Investigation, Data curation, Conceptualization. **Ming Ding:** Writing – original draft, Data curation, Conceptualization. **Hui Ding:** Writing – original draft, Data curation, Conceptualization. **Yuyan Qian:** Writing – review & editing, Supervision, Funding acquisition. **Jiaxing Hu:** Writing – original draft, Visualization. **Jukun Song:** Writing – review & editing, Supervision, Software. **Zhu Chen:** Writing – review & editing, Supervision, Methodology, Investigation, Formal analysis, Data curation, Conceptualization.

## Declaration of competing interest

The authors declare that they have no known competing financial interests or personal relationships that could have appeared to influence the work reported in this paper.
