# Supplementary material for: Understanding the consequences of leisure sedentary behavior on periodontitis: A two-step, multivariate Mendelian randomization study
Source: Heliyon. 2023 Nov 30;9(12):e23118. doi: 10.1016/j.heliyon.2023.e23118 (PMC10746448; doi:10.1016/j.heliyon.2023.e23118)

**Figure S1:** The flow chart of obtaining Instrumental variables（A, B, and C are based on the periodontitis data of the GLIDE Alliance； D, E, and F are based on periodontitis data from the Finnish database）

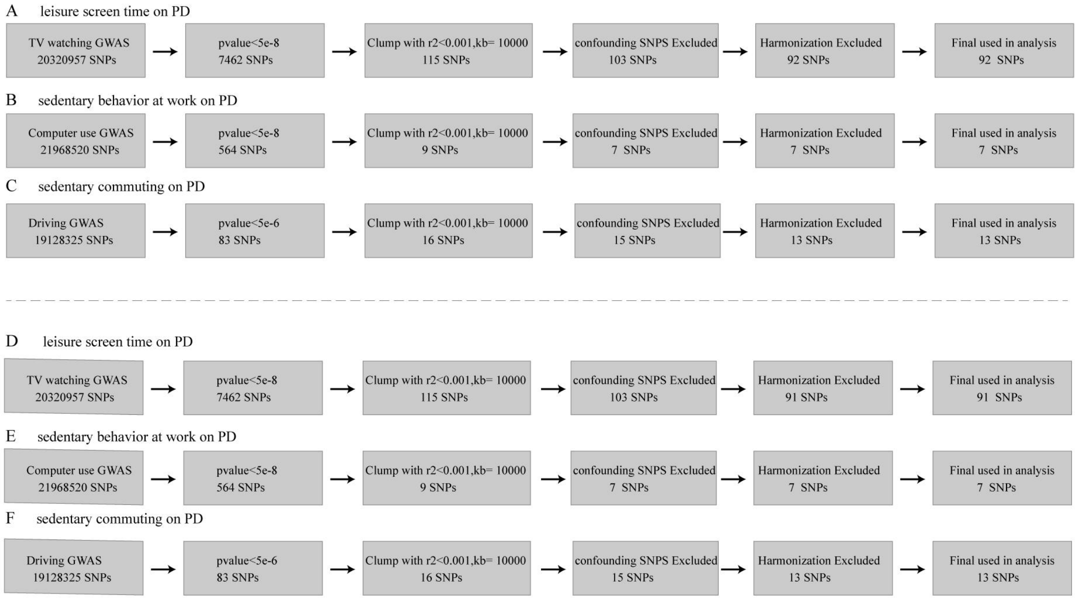


Abbreviation: **GWAS:** Genome-wide association studies, **SNP:** Single nucleotide polymorphism.

**Figure S2：** Scatter plots presenting the relationship between leisure screen time and Periodontitis from the GLIDE Alliance.

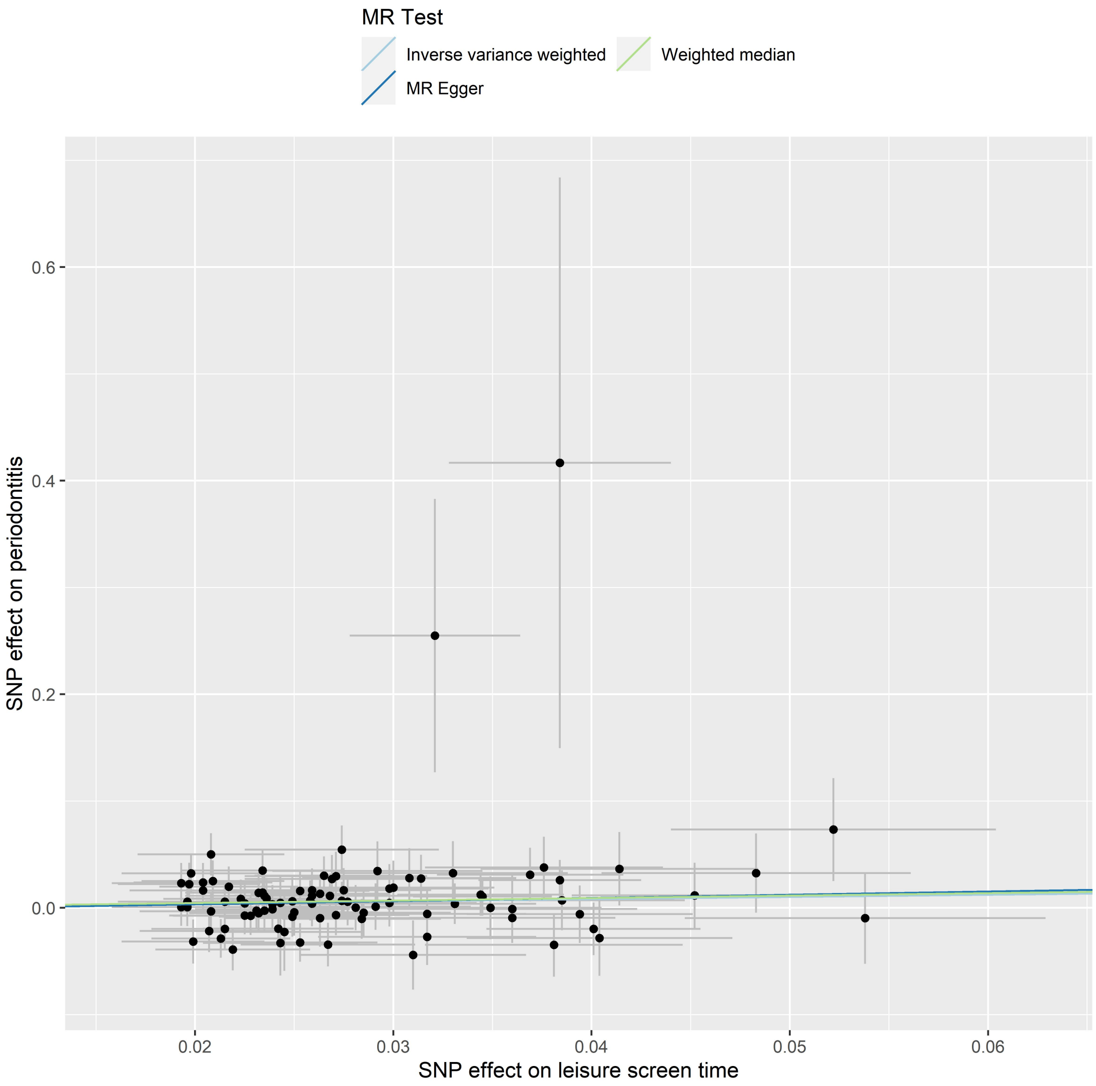

**Figure S3：** Leave-one-out plot presenting the relationship between leisure screen time and Periodontitis from the GLIDE Alliance.

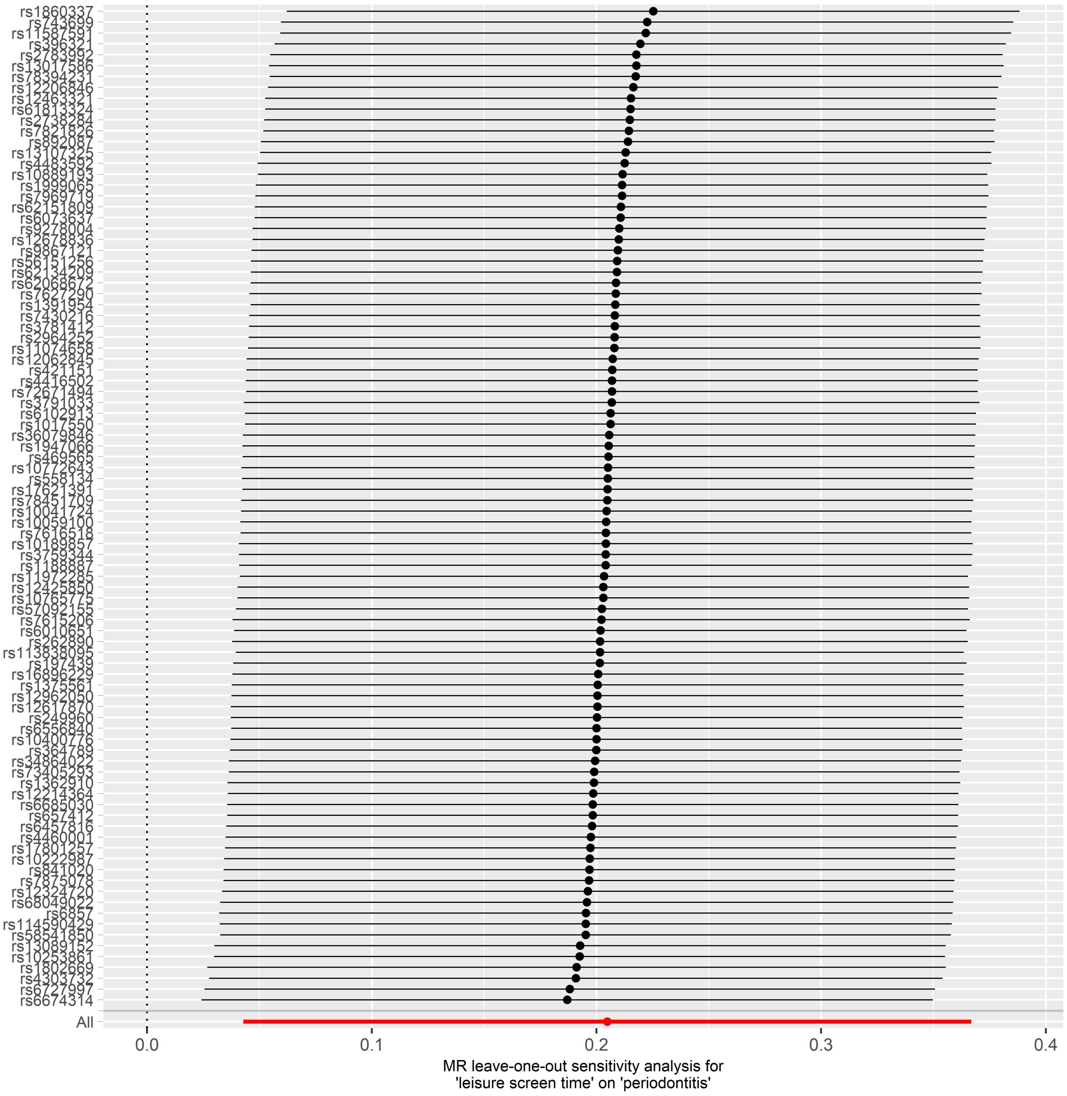

**Figure S4:** Funnel plots presenting the relationship between leisure screen time and Periodontitis from the GLIDE Alliance.

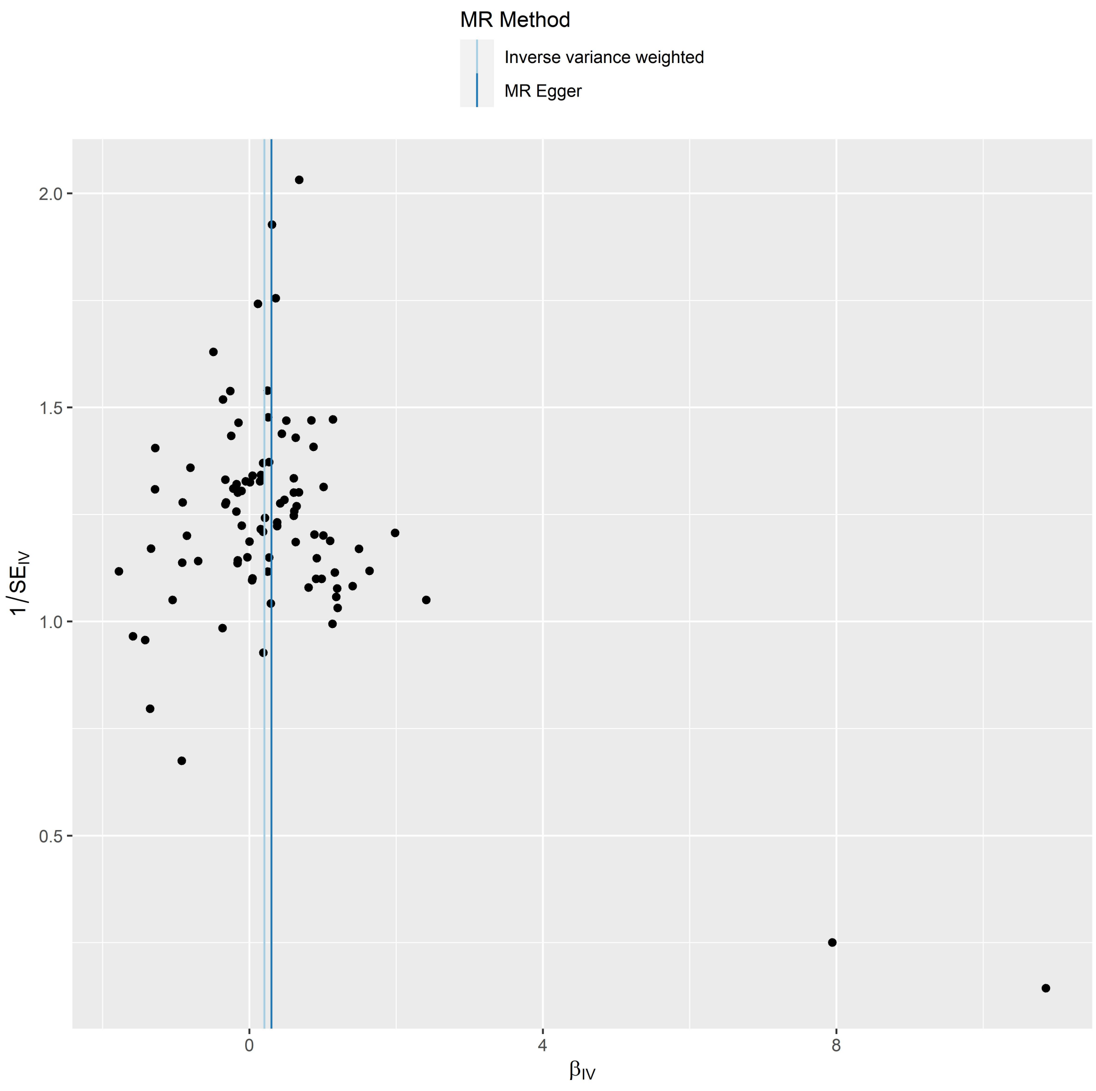

**Figure S5：** Scatter plot presenting the relationship between sedentary behavior at work and Periodontitis from the GLIDE Alliance.

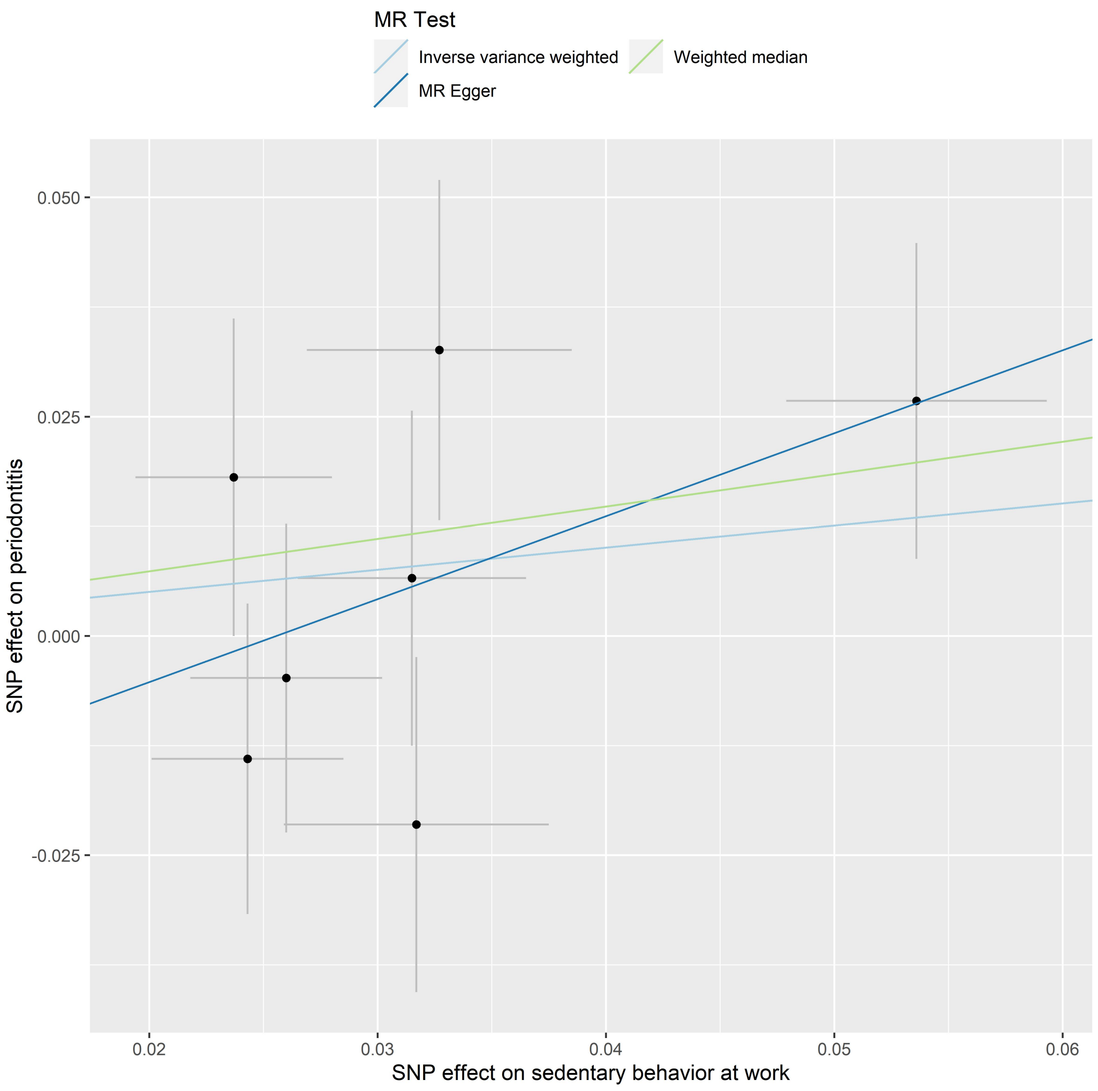

**Figure S6：** Leave-one-out plot presenting the relationship between sedentary behavior at work and Periodontitis from the GLIDE Alliance.

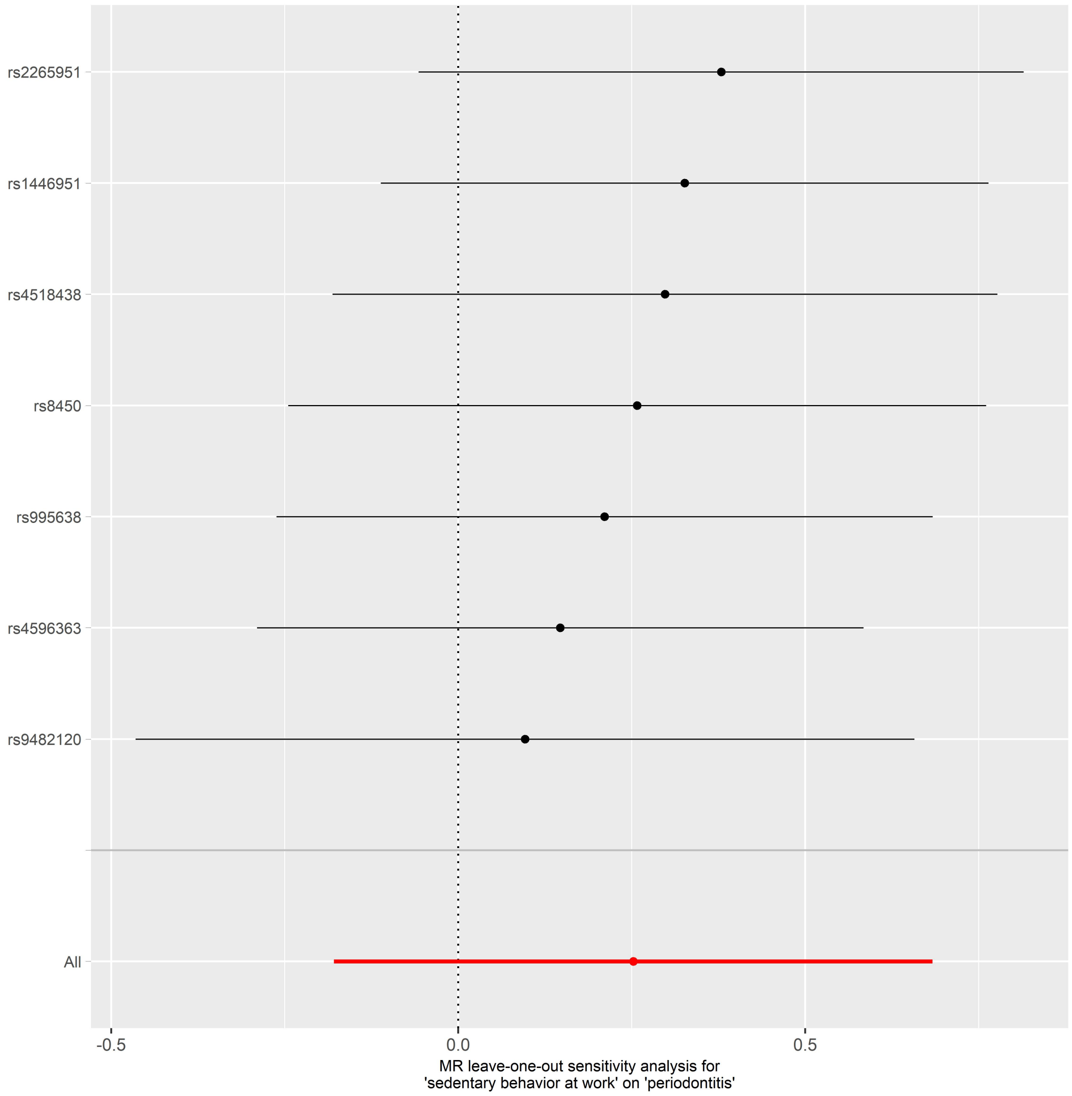

**Figure S7：** Funnel plot presenting the relationship between sedentary behavior at work and Periodontitis from the GLIDE Alliance.

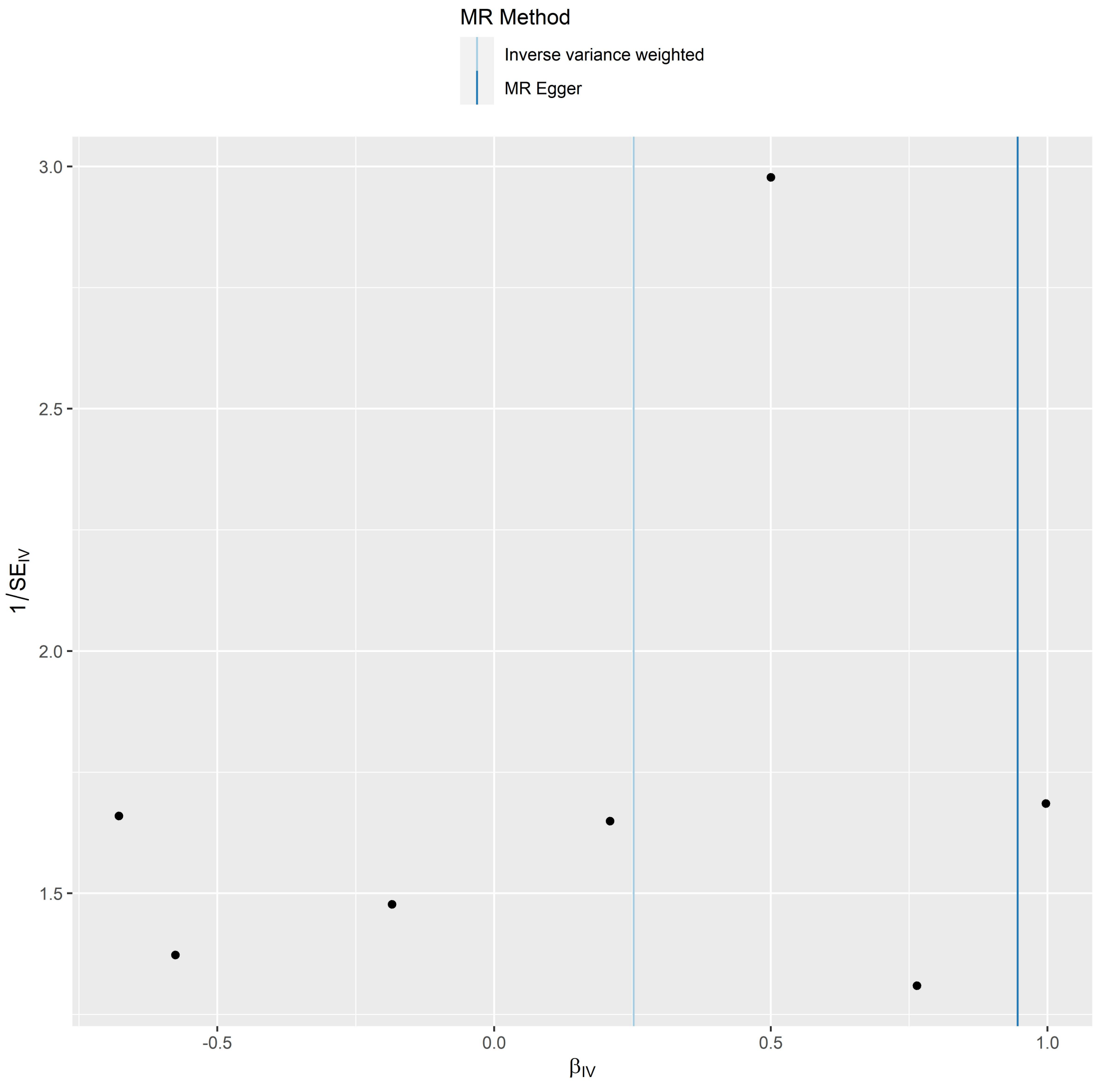

**Figure S8 :** Scatter plot presenting the relationship between sedentary commuting and Periodontitis from the GLIDE Alliance.

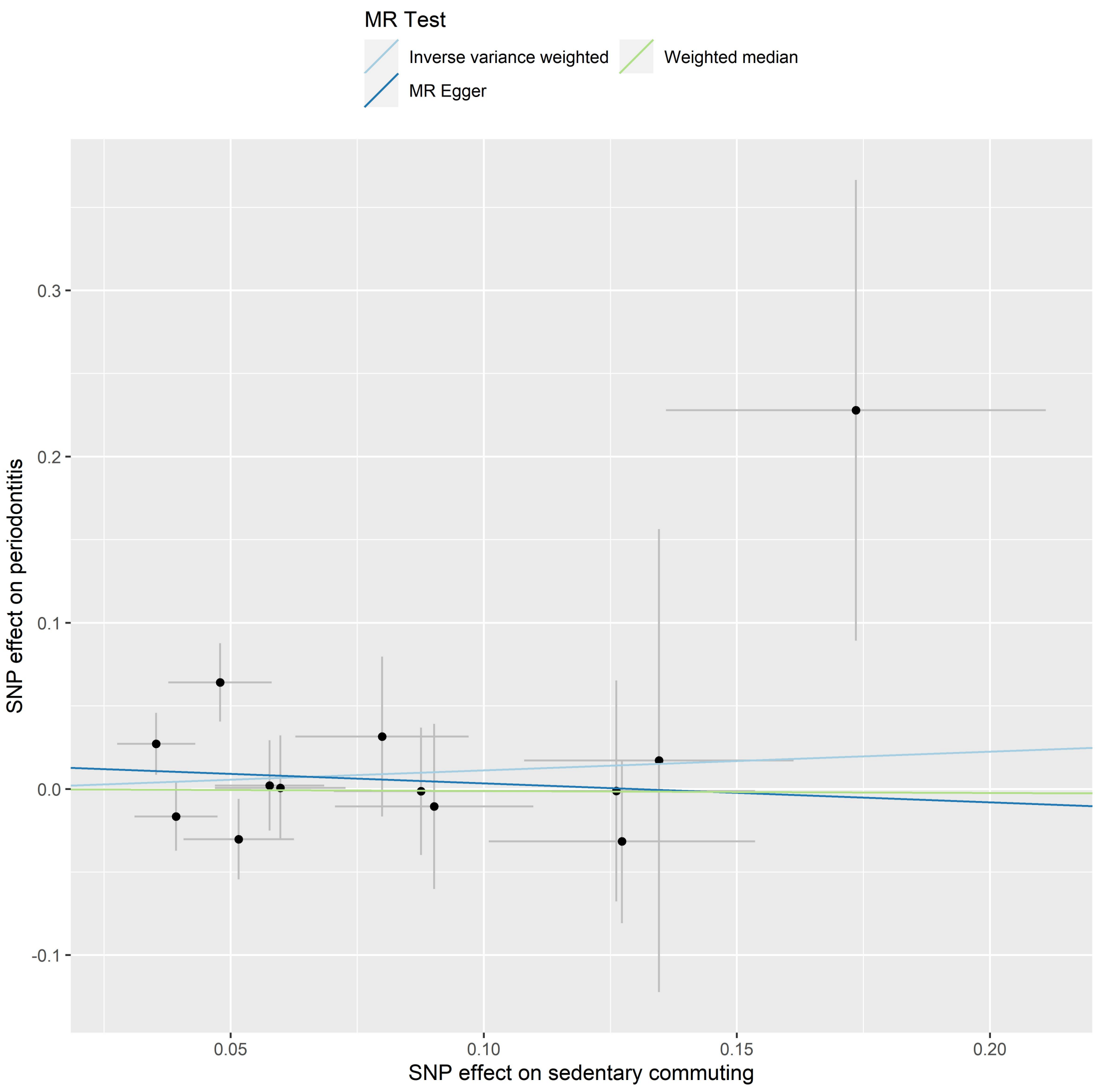

**Figure S9:** Leave-one-out plot presenting the relationship between sedentary commuting and Periodontitis from the GLIDE Alliance.

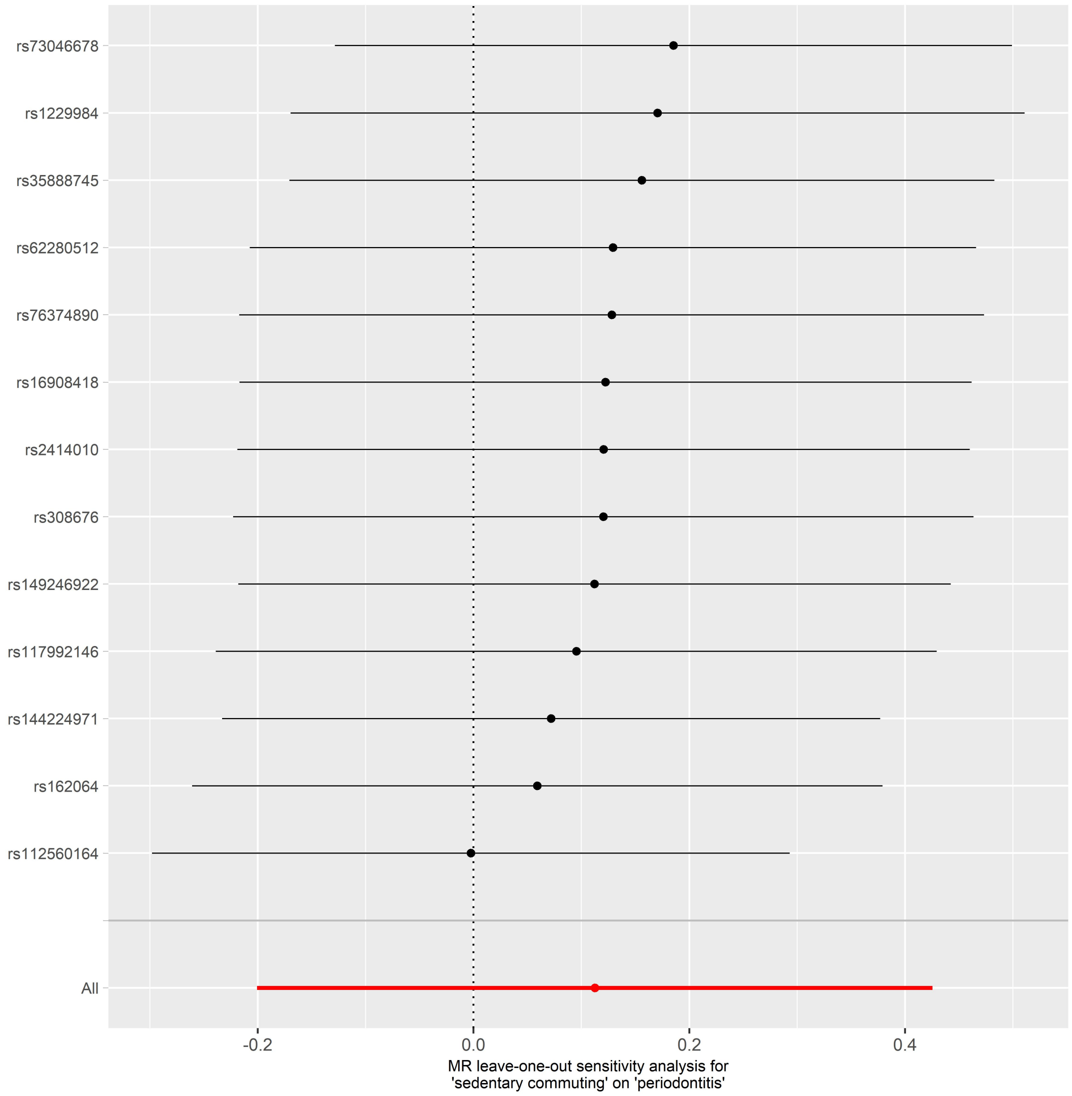

**Figure S10:** Funnel plot presenting the relationship between sedentary commuting and Periodontitis from the GLIDE Alliance.

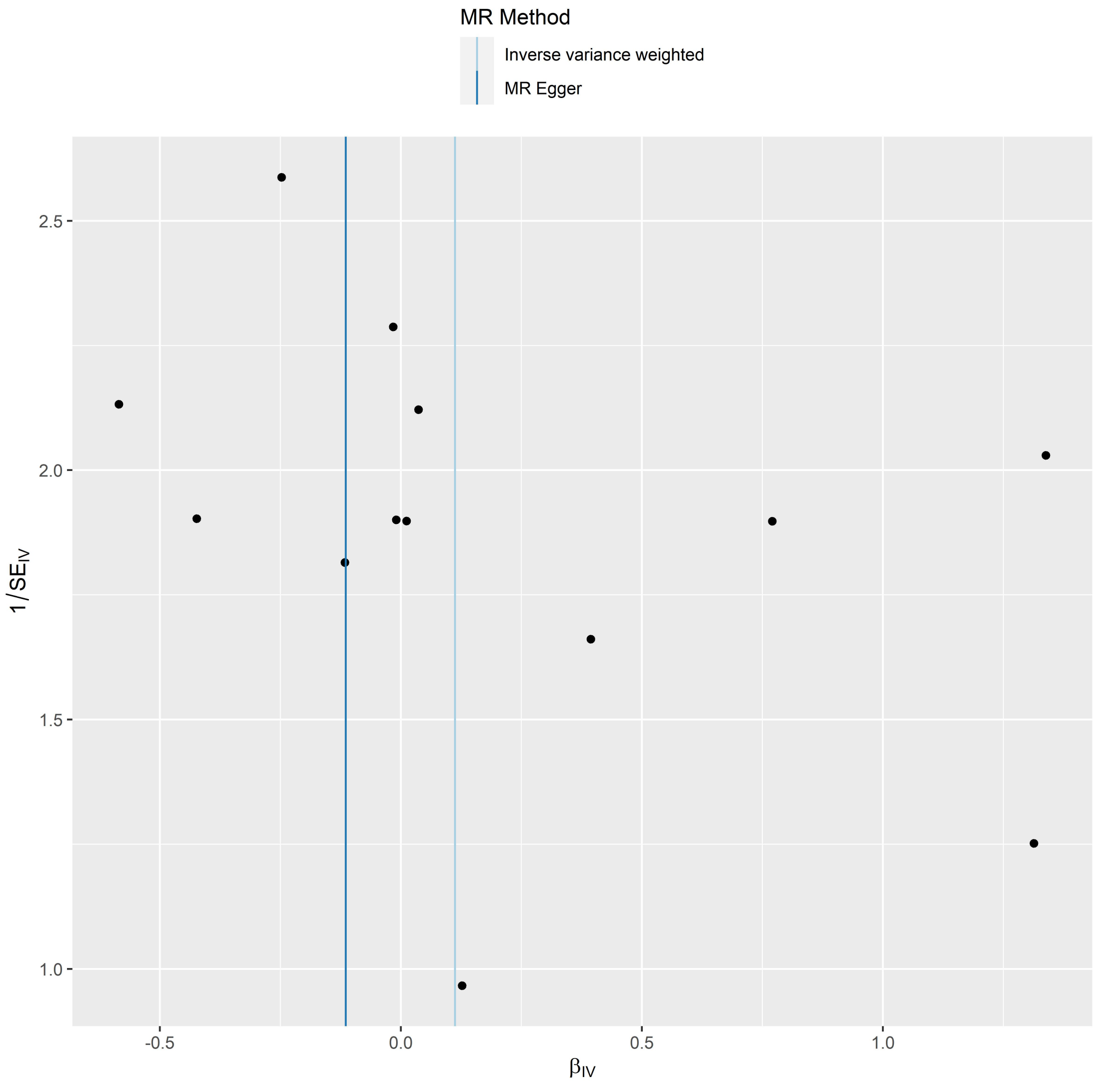

**Figure S11:** Scatter plots presenting the relationship between leisure screen time and Periodontitis from the FinnGen database.

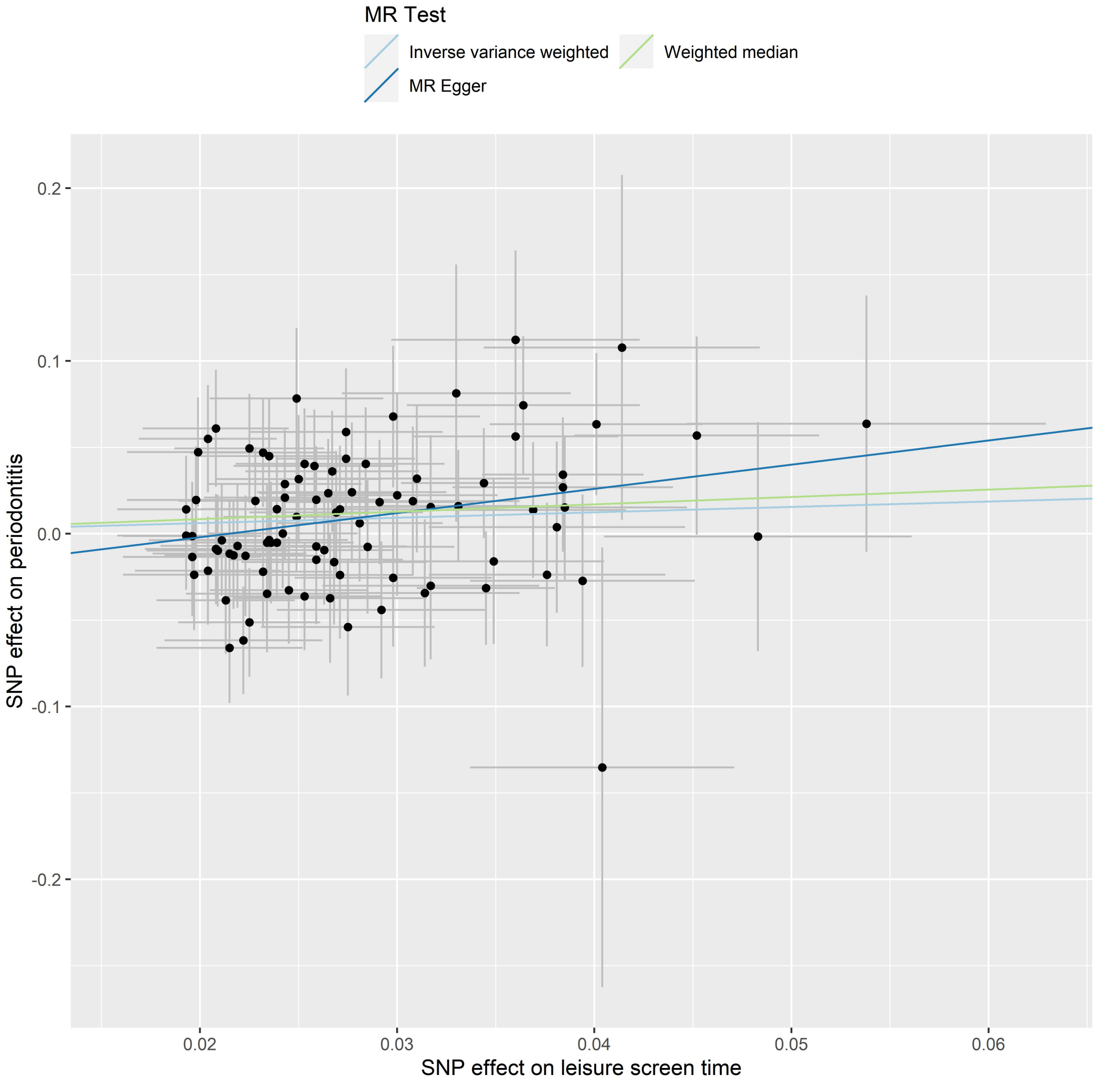

**Figure S12:** Leave-one-out plot presenting the relationship between leisure screen time and Periodontitis from the FinnGen database.

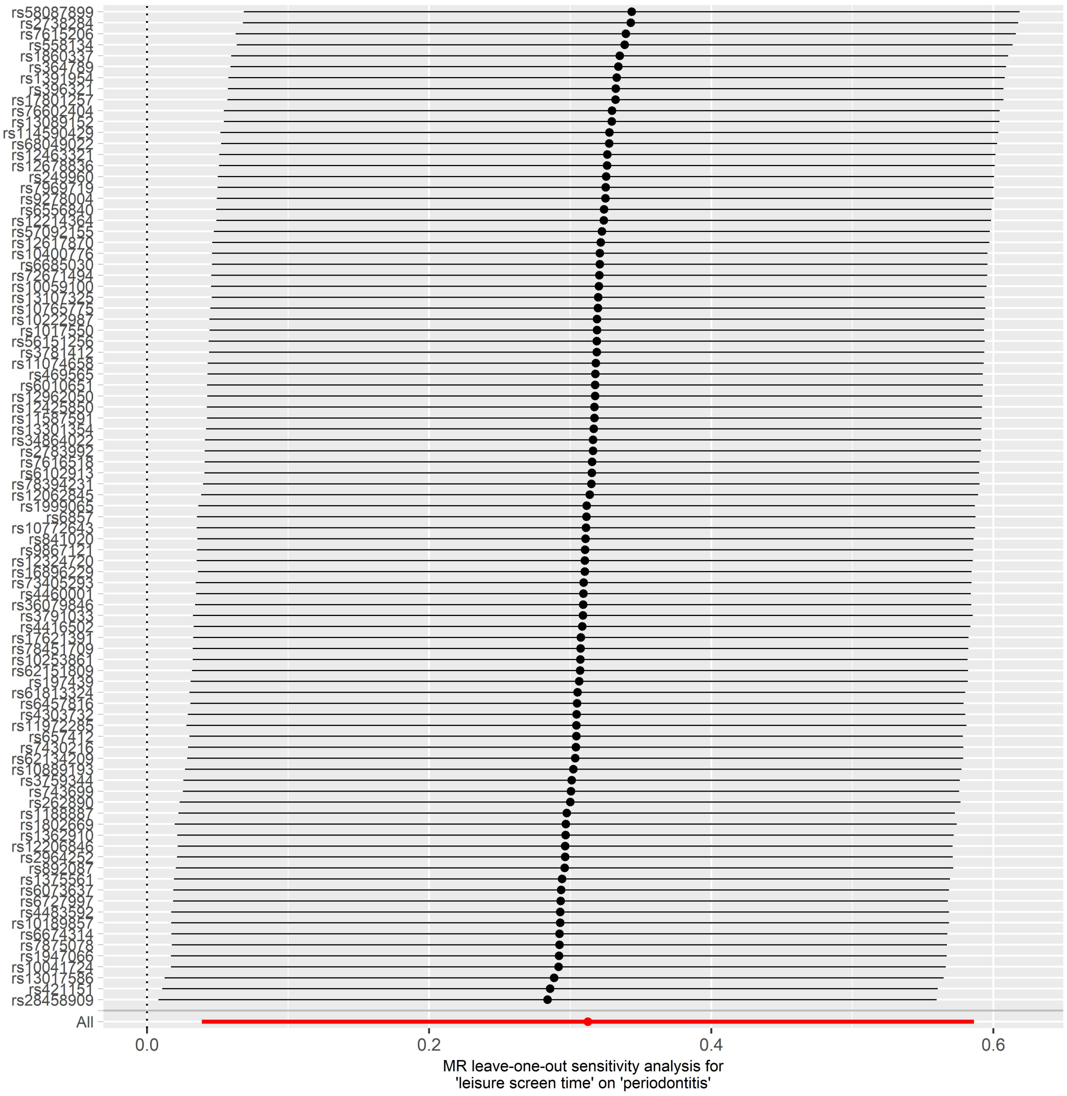

**Figure S13:** Funnel plots presenting the relationship between leisure screen time and Periodontitis from the FinnGen database.

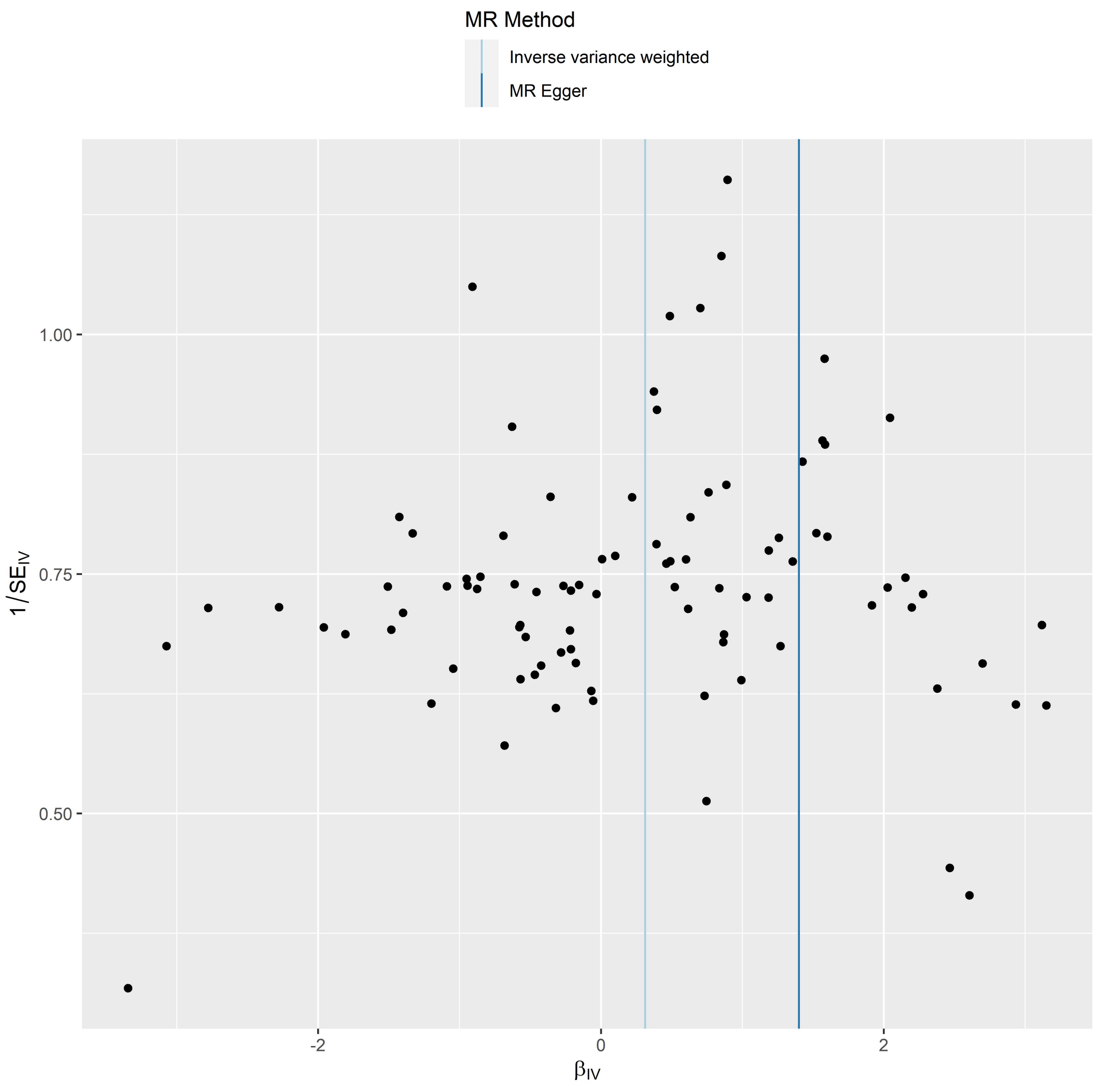

**Figure S14:** Scatter plot presenting the relationship between sedentary behavior at work and Periodontitis from the FinnGen database.

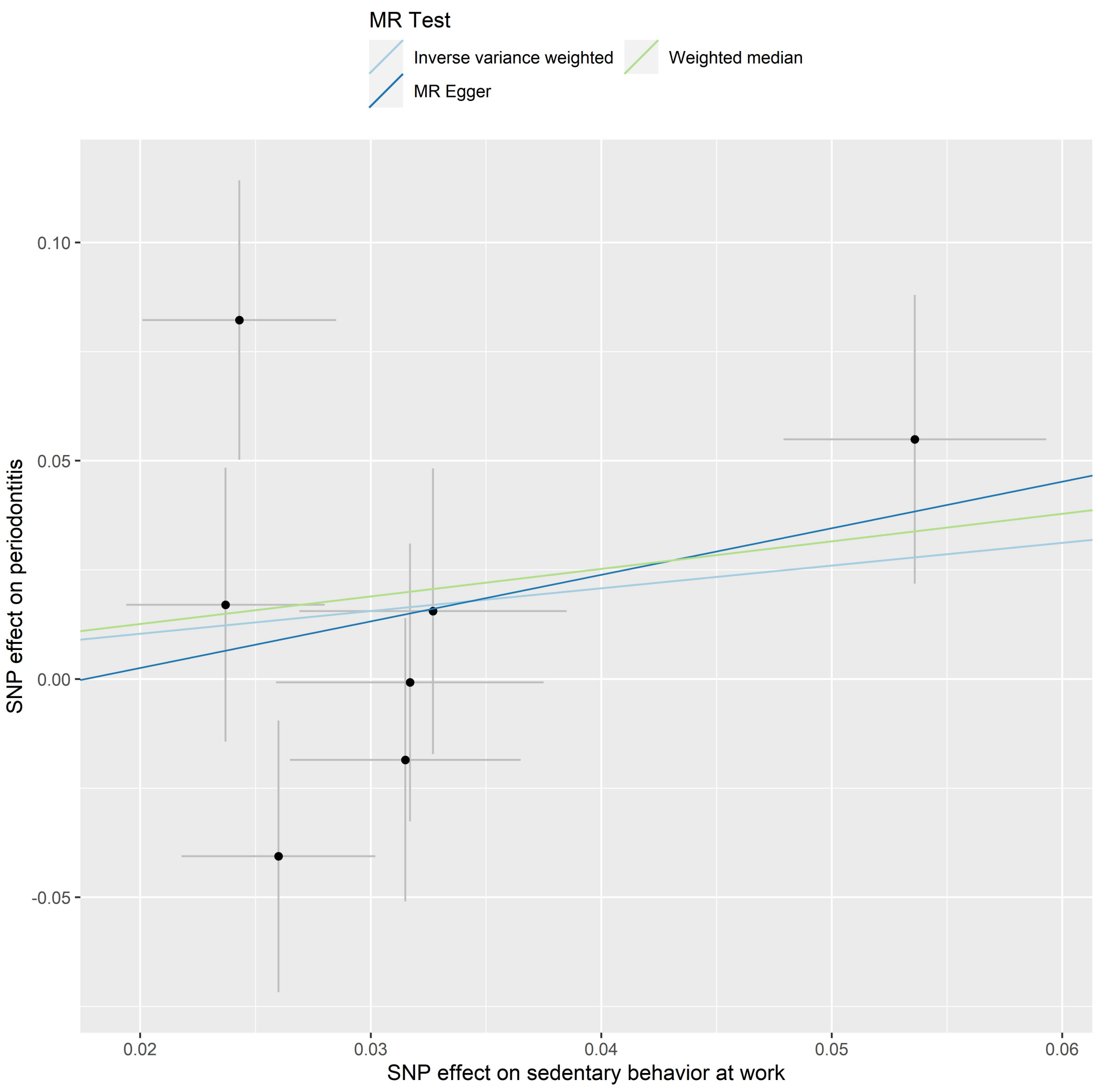

**Figure S15:** Leave-one-out plot presenting the relationship between sedentary behavior at work and Periodontitis from the FinnGen database.

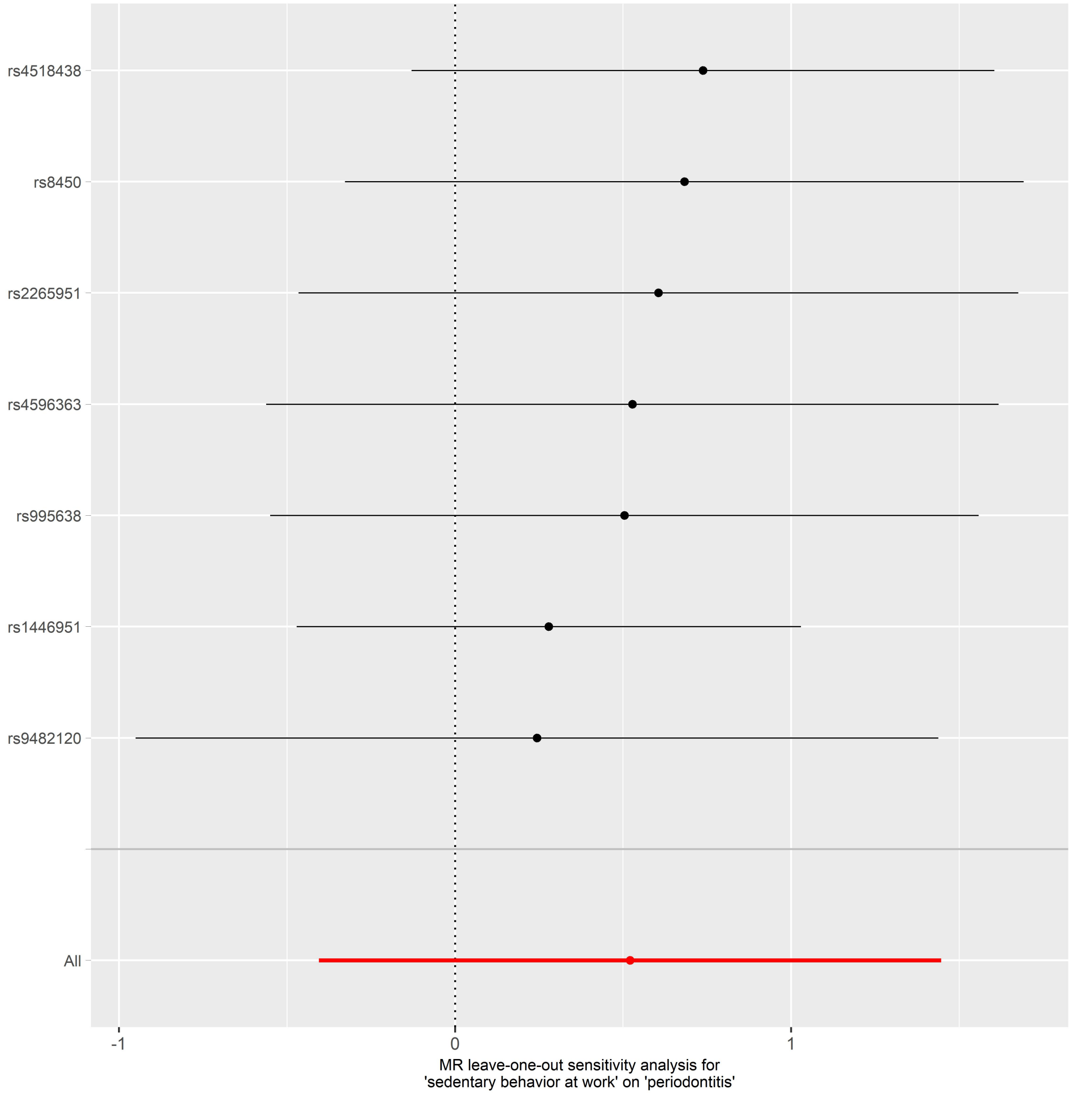

**Figure S16:** Funnel plot presenting the relationship between sedentary behavior at work and Periodontitis from the FinnGen database.

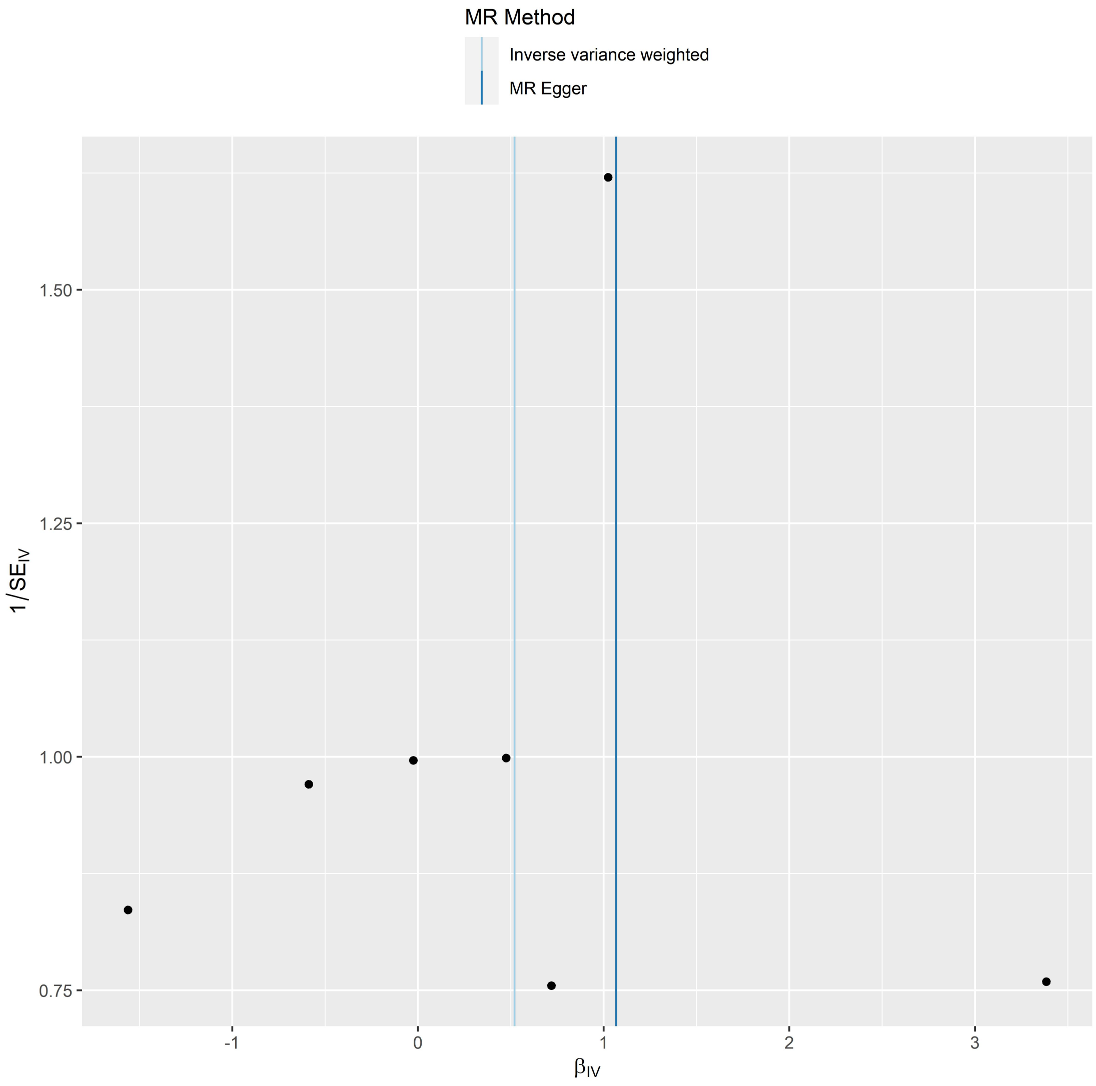

**Figure S17:** Scatter plot presenting the relationship between sedentary commuting and Periodontitis from the FinnGen database.

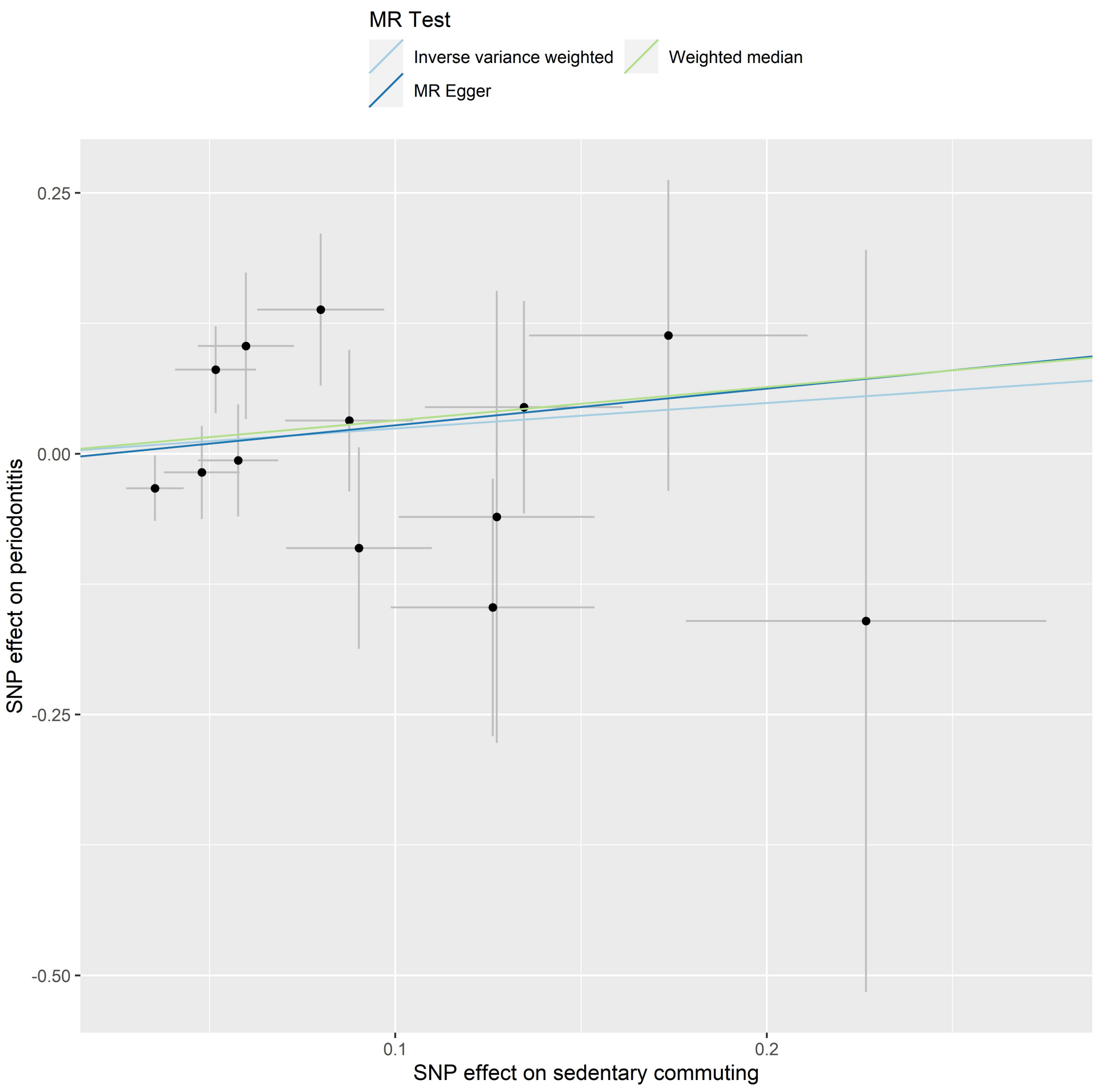

**Figure S18:** Leave-one-out plot presenting the relationship between sedentary commuting and Periodontitis from the FinnGen database.

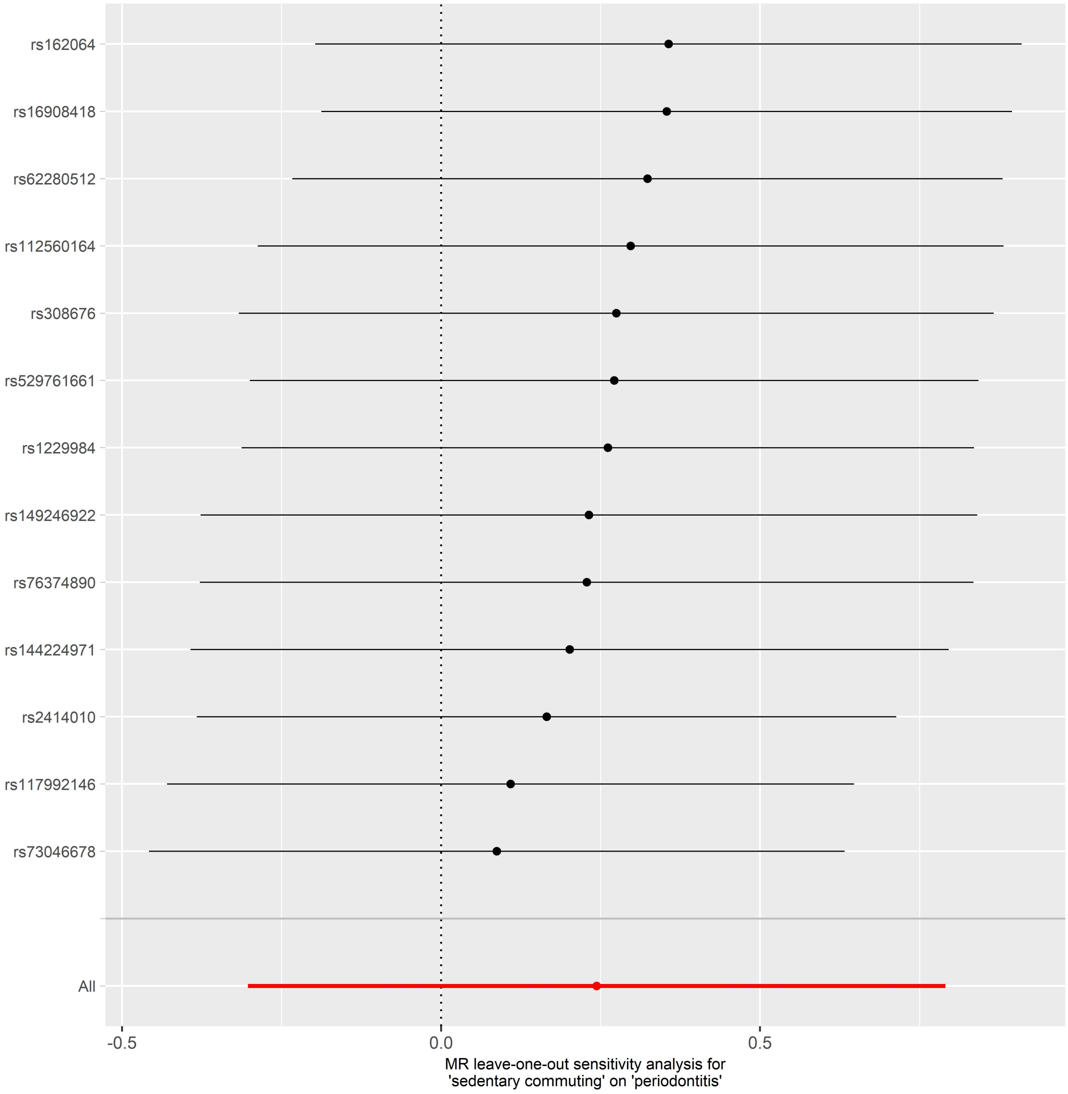

**Figure S19:** Funnel plot presenting the relationship between sedentary commuting and Periodontitis from the FinnGen database.

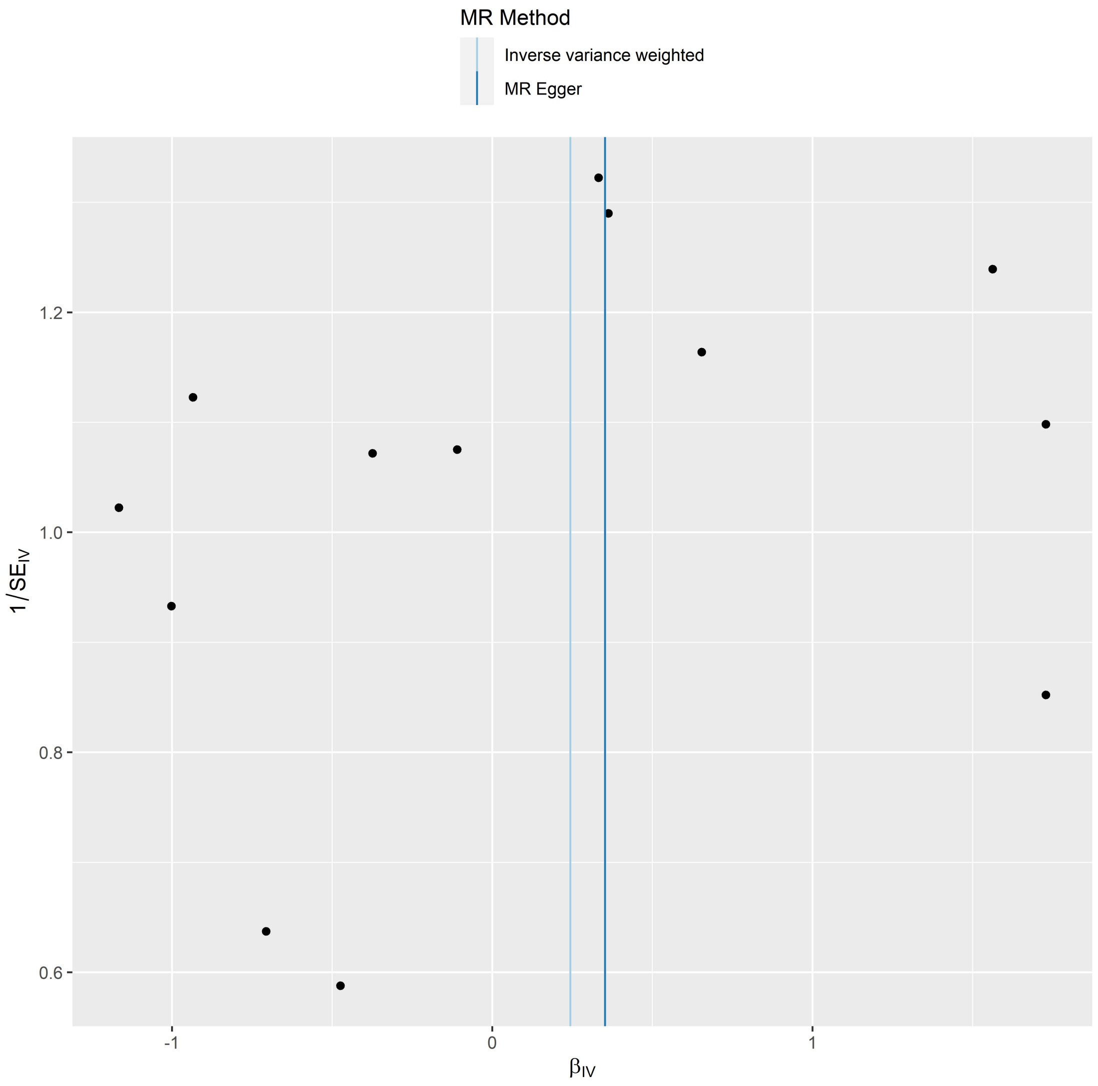

Supplement: Multimedia component 1 [file mmc1.pdf]
