# Supplementary material for: Understanding the consequences of leisure sedentary behavior on periodontitis: A two-step, multivariate Mendelian randomization study
Source: Heliyon. 2023 Nov 30;9(12):e23118. doi: 10.1016/j.heliyon.2023.e23118 (PMC10746448; doi:10.1016/j.heliyon.2023.e23118)
Supplement: Multimedia component 2 [file mmc2.docx]

**Table 1** Summary of Leisure sedentary behavior and periodontitis genome-wide association study

| **Trait** | **The first author** | **PMID/DOI** | **Sample size** | **Ancestry** | **Web source** |
| --- | --- | --- | --- | --- | --- |
| **leisure screen**  **time** | Z. Wang et al | 36071172 | 526,725 | European | <https://www.ebi.ac.uk/gwas/downloads/summary-statistics> |
| **sedentary commuting** | Z. Wang et al | 36071172 | 159,606 | European | <https://www.ebi.ac.uk/gwas/downloads/summary-statistics> |
| **sedentary behavior at work** | Z. Wang et al | 36071172 | 372,605 | European | <https://www.ebi.ac.uk/gwas/downloads/summary-statistics> |
| **periodontitis** | Shungin et al., 2019 | 31235808 | 34,615 | European | <https://doi.org/10.5523/bris.2j2rqgzedxlq02oqbb4vmycnc2> |
| **periodontitis** | Kurki et al., 2022 | https://doi.org/10.1101/2022.03.03.22271360 | 342,499 | European | <https://r8.finngen.fi/pheno/K11_PERIODON_CHRON_COMPL> |

**Table 2**  Summary of mediation trait genome-wide association Study

| **Trait** | **Trait** | **PMID** | **Sample size** | **Ancestry** | **Web source** |
| --- | --- | --- | --- | --- | --- |
| **Three types of Body fat distribution** | HiP | 25673412 | 224,459 | European | <https://portals.broadinstitute.org/collaboration/giant/index.php/GIANT_consortium_data_files> |
|  | WC | 25673412 | 224,459 | European | <https://portals.broadinstitute.org/collaboration/giant/index.php/GIANT_consortium_data_files> |
|  | WHP | 25673412 | 224,459 | European | <https://portals.broadinstitute.org/collaboration/giant/index.php/GIANT_consortium_data_files> |
| **BMI** | BMI | 30239722 | 806,834 | European | <https://portals.broadinstitute.org/collaboration/giant/index.php/GIANT_consortium_data_files> |
| **Five types of lipid symptoms** | TC | 34887591 | 884,255 | European | <https://csg.sph.umich.edu/willer/public/glgc-lipids2021/> |
|  | TG | 34887591 | 884,255 | European | <https://csg.sph.umich.edu/willer/public/glgc-lipids2021/> |
|  | HDL | 34887591 | 884,255 | European | <https://csg.sph.umich.edu/willer/public/glgc-lipids2021/> |
|  | LDL | 34887591 | 884,255 | European | <https://csg.sph.umich.edu/willer/public/glgc-lipids2021/> |
|  | nonHDL | 34887591 | 884,255 | European | <https://csg.sph.umich.edu/willer/public/glgc-lipids2021/> |
| **four blood sugar symptoms** | FG | 34059833 | 281,416 | European | <https://magicinvestigators.org/> |
|  | FI | 34059833 | 281,416 | European | <https://magicinvestigators.org/> |
|  | 2hGlu | 34059833 | 281,416 | European | <https://magicinvestigators.org/> |
|  | HbA1c | 34059833 | 281,416 | European | <https://magicinvestigators.org/> |
| **Drinking** | alcohol consumption | [30643251](https://pubmed.ncbi.nlm.nih.gov/30643251) | 941,280 | European | <https://www.nature.com/articles/s41467-020-18489-3> |
| **smoking** | tobacco smoking | [30643251](https://pubmed.ncbi.nlm.nih.gov/30643251) | 334,23 | European | <https://www.nature.com/articles/s41467-020-18489-3> |
