# Supplementary material for: Understanding the consequences of leisure sedentary behavior on periodontitis: A two-step, multivariate Mendelian randomization study
Source: Heliyon. 2023 Nov 30;9(12):e23118. doi: 10.1016/j.heliyon.2023.e23118 (PMC10746448; doi:10.1016/j.heliyon.2023.e23118)
Supplement: Multimedia component 3 [file mmc3.docx]

**Table S2** Summary of each Mendelian randomization method

| **MR method** | **Strengths and weaknesses** |
| --- | --- |
| IVW | Through a meta-analysis of the Wald estimates of each SNP, IVW obtains the overall estimate of the impact of exposure on the outcome, which has the best statistical performance under the assumption that all IVs have no multiplicity. |
| MR-Egger | MR-Egger is a statistical analysis based on the assumption that all IVs have multiple effects. MR-Egger has the most stringent assumptions for Instrumental variables, and the result shows that the confidence interval of its analysis results is wide. Because of its low statistical efficiency, it is more used to judge whether there is heterogeneity or not. |
| Weighted median | The weighted median method is a statistical analysis based on the assumption that half of the IVs are multiple. If the assumptions are met, it can provide unbiased estimates, but the efficiency may be low. |
| MR-RAPS | MR uses robust adjustment profile scores. Consider weak tool deviations, multiple effects, and extreme outliers. As long as the multiplicity is balanced, all SNP can be invalidated because of the multiplicity. At the same time, an unbiased estimation can be carried out in the presence of a large number of weak Instrumental variables. |
| MR-PRESSO | It is mainly used to judge whether there is an Instrumental variable with multiple effects and to remove potential Instrumental variables with multiple effects. |

**Abbreviations: IV**: instrumental variable; **MR**: Mendelian randomization; **IVW:** Inverse-variance weighted**; MR-RAPS:** Mendelian Randomization using a Robust Adjusted Profile Score**; MR-PRESSO**: Mendelian Randomization Pleiotropy RESidual Sum and Outlier; **SNP**: single nucleotide polymorphism.
