# Supplementary material for: Understanding the consequences of leisure sedentary behavior on periodontitis: A two-step, multivariate Mendelian randomization study
Source: Heliyon. 2023 Nov 30;9(12):e23118. doi: 10.1016/j.heliyon.2023.e23118 (PMC10746448; doi:10.1016/j.heliyon.2023.e23118)
Supplement: Multimedia component 5 [file mmc5.docx]

**Table S4：** The results of the evaluation of the statistical power of the MR model

| **Exposure** | **Outcome** | **Nsnp** | **F-statistic** | **power** | **Cochrane's Q** | **Intercept_P_value** | **Global Test** |
| --- | --- | --- | --- | --- | --- | --- | --- |
| **leisure screen time** | **PD** | 92 | **174** | 0.9 | 82 | 0.8 | 0.42 |
| **sedentary behavior at work** | **PD** | 7 | **112** | 0.37 | 5 | 0.34 | 0.38 |
| **sedentary commuting** | **PD** | 13 | **144** | 0.38 | 14 | 0.22 | 0.34 |
| **leisure screen time** | **PD from validation** | 91 | **171** | 0.81 | 80 | 0.09 | 0.59 |
| **sedentary behavior at work** | **PD from validation** | 7 | **112** | 0.56 | 9 | 0.08 | 0.25 |
| **sedentary commuting** | **PD from validation** | 13 | **144** | 0.77 | 13 | 0.26 | 0.47 |
