# Supplementary material for: Understanding the consequences of leisure sedentary behavior on periodontitis: A two-step, multivariate Mendelian randomization study
Source: Heliyon. 2023 Nov 30;9(12):e23118. doi: 10.1016/j.heliyon.2023.e23118 (PMC10746448; doi:10.1016/j.heliyon.2023.e23118)
Supplement: Multimedia component 6 [file mmc6.docx]

**Table S5：**Reverse MR analysis results

| **Exposure** | **Outcome** | **Nsnp** | **Method** | **beta** | **OR** | **95%CI** | **p_value** | **Heterogeneity P_value** | **Intercept_P_value** | **Global Test** |
| --- | --- | --- | --- | --- | --- | --- | --- | --- | --- | --- |
| **PD** | **leisure screen time** | 7 | **IVW** | 0.016 | 1.01 | (0.99, 1.04) | 0.19 | 0.32 | NA | NA |
|  |  |  | **MR–Egger** | 0.017 | 1.01 | (0.97, 1.06) | 0.47 | 0.46 | 0.97 | NA |
|  |  |  | **WM** | 0.027 | 1.02 | (0.99, 1.06) | 0.10 | NA | NA | NA |
|  |  |  | **MR-RAPS** | 0.016 | 1.01 | (0.99, 1.04) | 0.20 | NA | NA | NA |
|  |  |  | **MR-PRESSO** | 0.016 | 1.01 | (0.99, 1.04) | 0.19 | NA | NA | 0.361 |
| **PD from validation** | **leisure screen time** | 5 | **IVW** | 0.016 | 1.02 | (1.04, 1.02) | 0.14 | 0.9 | NA | NA |
|  |  |  | **MR–Egger** | 0.015 | 1.02 | (1.08, 1.02) | 0.67 | 0.96 | 0.99 | NA |
|  |  |  | **WM** | 0.019 | 1.02 | (1.04, 1.02) | 0.14 | NA | NA | NA |
|  |  |  | **MR-RAPS** | 0.016 | 1.02 | (1.03, 1.02) | 0.09 | NA | NA | NA |
|  |  |  | **MR-PRESSO** | 0.016 | 1.02 | (1.03, 1.02) | 0.14 | NA | NA | 0.462 |
| **smoking** | **leisure screen time** | 20 | **IVW** | -0.003 | 1 | (0.95, 1.05) | 0.91 | <0.01 | NA | NA |
|  |  |  | **MR–Egger** | -0.088 | 0.92 | (0.85, 0.99) | 0.03 | <0.01 | 0.09 | NA |
|  |  |  | **WM** | -0.034 | 0.97 | (0.93, 1.01) | 0.11 | NA | NA | NA |
|  |  |  | **MR-RAPS** | 0.007 | 1.01 | (0.98, 1.04) | 0.60 | NA | NA | NA |
|  |  |  | **MR-PRESSO** | -0.004 | 0.99 | (0.95,1.05) | 0.89 | NA | NA | 0.336 |
| **BMI** | **leisure screen time** | 451 | **IVW** | 0.345 | 1.41 | (1.35, 1.47) | **9.7E-56** | 0.22 | NA | NA |
|  |  |  | **MR–Egger** | 0.185 | 1.2 | (1.07, 1.36) | **2.7E-03** | 0.26 | 0.56 | NA |
|  |  |  | **WM** | 0.291 | 1.34 | (1.28, 1.40) | **5.5E-39** | NA | NA | NA |
|  |  |  | **MR-RAPS** | 0.353 | 1.42 | (1.39, 1.45) | **4.2E-71** | NA | NA | NA |
|  |  |  | **MR-PRESSO** | 0.325 | 1.38 | (1.30,1.46) | **2.4E-27** | NA | NA | 0.541 |
